# Hierarchical Metal‐Organic Framework Films with Controllable Meso/Macroporosity

**DOI:** 10.1002/advs.202002368

**Published:** 2020-11-13

**Authors:** Renheng Bo, Mahdiar Taheri, Borui Liu, Raffaele Ricco, Hongjun Chen, Heinz Amenitsch, Zelio Fusco, Takuya Tsuzuki, Guihua Yu, Rob Ameloot, Paolo Falcaro, Antonio Tricoli

**Affiliations:** ^1^ Nanotechnology Research Laboratory Research School of Electrical, Energy, and Materials Engineering Australian National University Canberra 2601 Australia; ^2^ Laboratory of Advanced Nanomaterials for Sustainability Research School of Electrical, Energy, and Materials Engineering Australian National University Canberra 2601 Australia; ^3^ Institute of Physical and Theoretical Chemistry Graz University of Technology Stremayrgasse 9/Z2 Graz 2010 Austria; ^4^ Institute of Inorganic Chemistry Graz University of Technology Stremayrgasse 9/Z2 Graz 2010 Austria; ^5^ Materials Science and Engineering Program and Department of Mechanical Engineering The University of Texas at Austin Austin Texas 78712 USA; ^6^ Centre for Membrane Separations Adsorption, Catalysis, and Spectroscopy for Sustainable Solutions Leuven 3001 Belgium

**Keywords:** extrinsic porosity, metal‐organic frameworks, molecular sieving, 3D structuring

## Abstract

The structuring of the metal‐organic framework material ZIF‐8 as films and membranes through the vapor‐phase conversion of ZnO fractal nanoparticle networks is reported. The extrinsic porosity of the resulting materials can be tuned from 4% to 66%, and the film thickness can be controlled from 80 nm to 0.23 mm, for areas >100 cm^2^. Freestanding and pure metal‐organic frameworks (MOF) membranes prepared this way are showcased as separators that minimize capacity fading in model Li‐S batteries.

## Graphical abstract

A flexible strategy for three‐dimensional structuring of hierarchical metal‐organic frameworks monoliths is presented, which provides unique advantages, including: structural homogeneity, tunable extrinsic porosity, high scalability, and designable geometrical features. The superior control over metal‐organic frameworks’ morphologies and dimensions of the proposed conversion route enables the design and fabrication of a large family of hierarchical metal‐organic frameworks architectures.

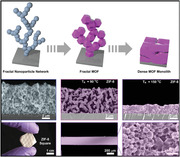

## 1 Introduction

Metal‐organic frameworks (MOFs) are extended materials consisting of inorganic nodes (metal clusters or ions) held together by multitopic organic linkers.^[^ ^1^, ^2^ ^]^ Their well‐defined pore size,^[^ ^2^ ^]^ tunable topology,^[^ ^3^ ^]^ high accessible surface area, and chemical mutability,^[^ ^4^ ^]^ make MOFs emerging materials for applications in gas storage,^[^ ^5^ ^]^ separation,^[^ ^6^ ^]^ catalysis,^[^ ^7^ ^]^ biotechnology,^[^ ^8^ ^]^ optics,^[^ ^9^ ^]^ microelectronics,^[^ ^10^ ^]^ and energy production/storage.^[^ ^11^, ^12^ ^]^ However, for each application, MOFs need to be obtained in a suitable shape. For instance, while for gas storage applications synthesis and shaping of bulk powders is the most viable approach, other applications require the fabrication of MOFs as supported films or self‐standing membranes. Moreover, while for the integration into microelectronics thin, smooth, and pinhole‐free films are preferred, other applications benefit from coatings with a controllable interparticle porosity to ensure rapid mass transport (e.g., solid‐phase microextraction^[^ ^13^ ^]^ or chromatography).^[^ ^14^ ^]^ Thus far, very few methods are available to deposit the latter type of mesoporous MOF films.^[^ ^15^ ^]^

**Figure 1:**
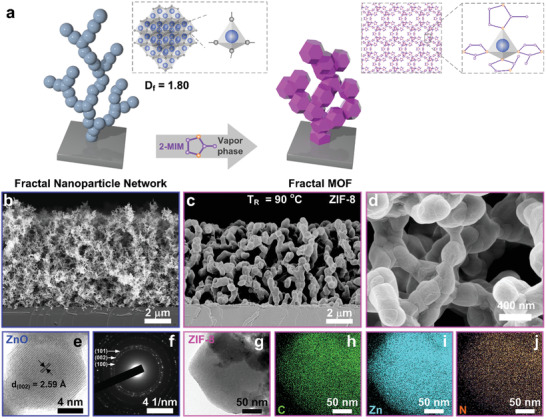
Hierarchical MOF films through the conversion of fractal nanoparticle networks. a) Schematic representation of the conversion of a ZnO fractal nanoparticle network to ZIF‐8, for a typical fractal dimension (*D*_f_) of the ZnO. b) SEM micrograph of a ZnO FNN and c,d) the resulting structured ZIF‐8. e,f) HRTEM image and electron diffraction pattern of a ZnO nanoparticle detached from the network in b. g‐j) HAABF‐STEM image of a ZIF‐8 particle detached from the network in**c**and elemental mapping. C, Zn, and N are represented in green, blue, and orange, respectively.

Here, we report a protocol that uses oxide fractal nanoparticle networks (FNNs) as precursors for the binder‐free shaping of MOF films. By tuning the oxide‐to‐MOF conversion, our approach enables to tailor the interparticle porosity. In general, the conversion of ceramic precursors to MOFs has shown to be an efficient route to fabricate films.^[^ ^16^, ^17^, ^18^ ^]^ For instance, Ameloot and co‐workers^[^ ^17^ ^]^ demonstrated that ZnO films could be converted into ZIF‐8 via exposure to a vapor of the 2‐methylimidazole (2‐MIM) linker. However, the conversion was limited to a depth of 15 nm from the top ZnO film surface. Instead of using dense oxide films, our approach leverages porous FNNs as 3D templates (**Figure** **1**). Because of their high porosity, exposure of these FNNs to 2‐MIM vapors results in their full conversion to MOF, while preserving the original fractal arrangement and advantages of solvent‐free synthesis.

## 2 Hierarchical MOF Films

FNNs were prepared by the self‐assembly of oxide precursor aerosols in the diffusion‐limited cluster‐cluster aggregation regime.^[^ ^19^ ^]^ This approach is a scalable and reproducible route for the generation of materials with a fractal dimension (*D*_f_) of 1.7–1.8.^[^ ^19^ ^]^ The thickness of the resulting film can be adjusted in the nano‐ to milli‐meter range and depends linearly on the deposition time.^[^ ^20^ ^]^ We used a liquid‐fed flame synthesis reactor that permits the continuous synthesis of a broad range of metal oxide aerosols at a production rate up to 1 kg h^−1^,^[^ ^21^ ^]^ and offers control over the nanoparticle size. Based on earlier reports,^[^ ^17^, ^18^ ^]^ we hypothesized that the particle size is a crucial parameter to achieve full ceramic‐to‐MOF conversion and optimized our method for the deposition of ZnO FNNs with primary particles smaller than 20 nm (Figure 1e,f S1, Supporting Information).^[^ ^22^, ^23^ ^]^ Observations of the conversion of these ZnO FNNs by in situ synchrotron SAXS revealed a higher reactivity to 2‐MIM compared to micrometer‐sized ZnO powders (Figure S2, Supporting Information). In addition, the open fractal network was crucial for the full conversion into MOF (Figure S3–7, Supporting Information). Only for ZnO FNNs with an extrinsic porosity ≥97%, full conversion into crack‐free ZIF‐8 films was observed (Figures S5–9, Supporting Information). For lower porosity fractions, the volume expansion that occurs during the oxide‐to‐MOF conversion (10–17 times, depending on the ZnO crystallinity)^[^ ^17^, ^24^ ^]^ limits the diffusion of the ligand, resulting in a partial conversion, residual stress, and cracks within the final MOF films (Figure S5, Equation 1, Supporting Information). The full conversion to ZIF‐8 was confirmed by the absence of ZnO diffraction peaks (Figures S6,7,10a, Supporting Information) and FTIR spectroscopy (Figure S10b, Supporting Information). High‐angle annular bright‐field scanning transmission electron microscopy (HAABF‐STEM) and elemental mapping confirmed the homogeneous distribution of carbon, zinc, and nitrogen in the ZIF‐8 particles (Figure 1g–j). SEM analysis revealed that the texture of the resulting ZIF‐8 films is dictated by the ZnO FNN precursor (Figures S11–13, Supporting Information) (Figure 1c,d). For instance, a ZnO FNN precursor with a fractal dimension (*D*_f_) of 1.8 and an accessible porosity of 98% yielded a ZIF‐8 film with 66% of extrinsic porosity (Figure 1b–d).

## 3 Tuning the MOF Film Structure and Thickness

**Figure 2:**
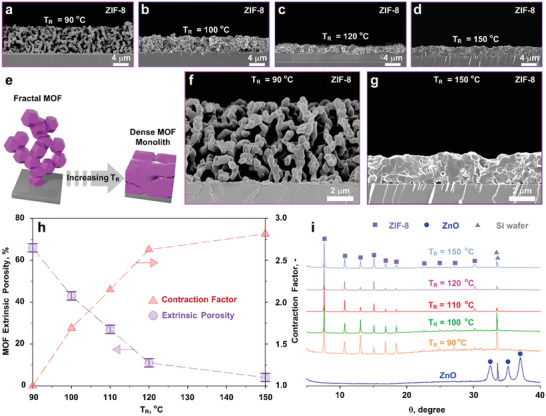
Tuning the extrinsic porosity of hierarchical ZIF‐8 films. Cross‐sectional SEM images of ZIF‐8 films with: a) 66% extrinsic porosity (*T*_R_= 90 °C); b) 43% extrinsic porosity (*T*_R_= 100 °C); c) 11% extrinsic porosity (*T*_R_= 120 °C); d) 4% extrinsic porosity (*T*_R_= 150 °C). e) Scheme of grain coalescence of ZIF‐8 crystals based on ZIF‐8 obtained at 90 and 150 °C (f,g, respectively). h) Correlation between the extrinsic ZIF‐8 porosity and the reaction temperature. i) XRD patterns of a ZnO FNN (dark blue line) and the resulting ZIF‐8 films as a function of the conversion temperature.

Our fractal‐to‐MOF conversion protocol allows to control the extrinsic porosity in the 4–66% range by controlling the reaction temperature (*T*_R_). **Figure** **2**a–d shows ZIF‐8 films obtained at 90–150 °C. At *T*_R_ = 90 °C, the thickness of the ZnO FNN precursor was maintained in the MOF film, and an extrinsic porosity of 66% was measured (Figure 2a, Figures S14 c–d, S15 and Table S1, Supporting Information). The increase in solid fraction (SF) from the precursor ZnO FNN (SF = 2%) to the ZIF‐8 film (SF = 34%) matches well with the 17 folds’ theoretical volume expansion from ZnO (wurtzite) to ZIF‐8.^[^ ^17^ ^]^ The compaction of the MOF films with increasing reaction temperatures was attributed to the coalescence of the MOF grains (Figure 2e). Increasing *T*_R_ from 90 to 100, 110, 120, and 150 °C, induced a progressive compaction and a reduction in the extrinsic porosity from 66% to 43%, 27%, 11%, and 4%, respectively (Figure 2a–d,f–g, Figures S15–19 and Table S1, Supporting Information). The extrinsic porosity decreases linearly with *T*_R_ below 120 °C (Figure 2h), with high reproducibility (Table S1, Supporting Information). Full ZnO conversion was observed irrespective of *T*_R_ (Figure 2i, Figure S10, Supporting Information).

The diffraction plot after 10 min conversion (flame‐made ZnO FNN in presence of 2‐MIM at 90 °C) shows a full conversion. By increasing the reaction time (18 h) of ZnO in presence of 2‐MIM at 90 °C we did not observe in any further densification of the ZIF‐8 films. Thus, the here reported nanoparticle network of 20 nm ZnO showed a similar reaction kinetics to the previously reported dense ZnO 10 nm films (i.e., 10–30 min vapor‐phase reaction for a full conversion into crystalline ZIF‐8, sod).^[^ ^17^ ^]^ In the reactor, we added an amount of ligand sufficient to maintain its saturation pressure at all temperatures for the ZnO conversion to ZIF‐8 (see Supporting Information). To examine the effect of the saturation pressure, we decreased the amount of 2‐MIM from 100 mg to 2 mg for the 150 °C conversion temperature. By our calculation, the pressure with 2 mg (*P*_2mg@150_ = 1070.90 Pa) is 42% the saturation pressure at 150 °C. With *P*_2mg@150_, after 18 h reaction time at 150 °C, we observed only a partial conversion of ZnO to ZIF‐8 (Figure S32, Supporting Information). Our experiments suggest that the conversion of ZnO to ZIF‐8 can be enhanced when the pressure of the ligand in the gas phase is equal, or higher, to its saturation pressure.

**Figure 3:**
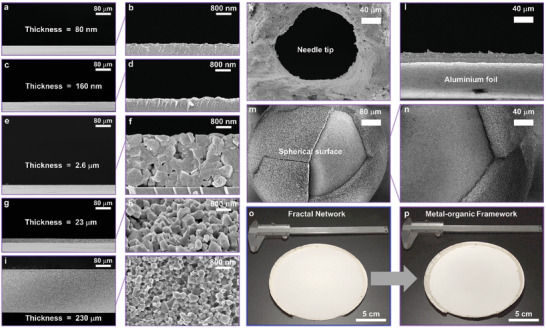
Controlling the ZIF‐8 film thickness range and versatile coatings. a–j) Representative cross‐sectional scanning electron microscope images of ZIF‐8 films with an extrinsic porosity of 4%, obtained by conversion of ZnO FNNs at 150 °C. The ZIF‐8 film thickness (80 nm to 230 µm) was controlled by tuning the amount of deposited ZnO. k) SEM image of a ZIF‐8 channel prepared using a template. l–n) Cross‐sectional SEM images of a ZIF‐8‐coated aluminum foil and sphere. o,p) Images of a ZnO precursor film deposited on a 177 cm^2^glass fiber, before and after conversion to ZIF‐8.

FNNs afford the fabrication of nanometer‐ to millimeter‐thick MOF films over areas >100 cm^2^. **Figure** **3** shows crack‐free ZIF‐8 films with a thickness ranging from 80 to 0.23 mm (Figure 3a–j, Figure S20, Supporting Information). Thermogravimetric and XRD analysis confirmed the complete conversion of the ZnO precursor (Figures S21–S22, Supporting Information). MOF coatings were fabricated on a needle (∅ = 550 µm), a ceramic milling ball (∅ = 1 mm), and flexible aluminum foils (Figure 3k–n, Figure S23, Supporting Information). The scalability of the method was illustrated by coating a 177 cm^2^ area with ZIF‐8 (Figure 3o,p).

## 4 Self‐Supporting MOF Membranes as Battery Separators

**Figure 4:**
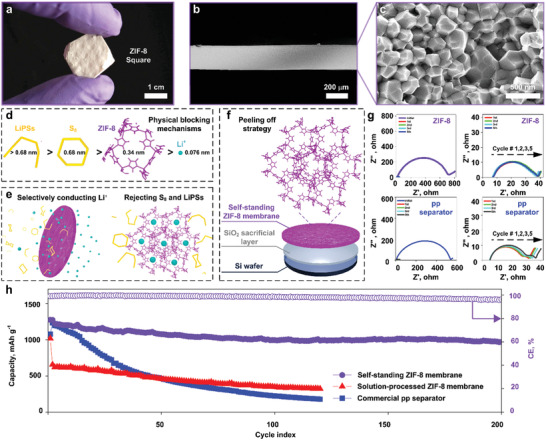
Self‐supporting ZIF‐8 membranes as battery separators. a–c) Optical and cross‐sectional SEM images of a self‐supporting square ZIF‐8 membrane. d,e) ZIF‐8 is expected to discriminate Li^+^and Li‐polysulfides based on their size. f) The peeling off strategy for the preparation of the self‐supporting ZIF‐8 separator (1.9 cm in diameter and *≈*23 µm in thickness). g) Ionic conductivity (Li^+^) and blocking effect for LiPSs characterized by electrochemical impedance spectroscopy (EIS). h) Efficient LiPS molecular sieving demonstrated by the improved cycling performance when the ZIF‐8 separator is used.

Typically, binderless MOF films are fragile and require specialized protocols to be transferred.^[^ ^18^ ^]^ To fabricate robust, self‐supporting MOF membranes, the ZnO FNN precursor was deposited on top of a sacrificial SiO_2_ FNN (Figure S24, Supporting Information). As the SiO_2_ particles are kept together only by weak van der Waals forces,^[^ ^25^ ^]^ minimal mechanical stress is required for the lift‐off of the MOF membrane. This approach could be used to fabricate self‐supporting ZIF‐8 membranes with a thickness ranging from 12 to 230 µm (**Figure** **4**a–c, Figure S24b–c,g–i, Supporting Information). Due to the ZnO FNN deposition rate (120 min for a 0.66 mm thick FNN on 177 cm^2^), we limited the precursor thickness. However, thicker MOF‐based components could be prepared at higher deposition rates.^[^ ^21^ ^]^

MOFs membranes can display selective ion conductivity.^[^ ^26^ ^]^ This property is highly desirable to realize the potential of Li‐S batteries, since Li^+^ should diffuse through the separator while the migration of Li‐polysulfides (LiPSs) should be prevented. Based on its 0.34 nm pore aperture, ZIF‐8 (sodalite topology) should be able to discriminate between Li^+^ (0.076 nm) and long‐chain LiPSs (>0.68 nm) (Figure 4d,e), even when taking into account the framework flexibility.^[^ ^27^ ^]^ Existing MOF fabrication protocols did not yield a crack‐free membrane.^[^ ^28^ ^]^ Therefore, self‐supporting ZIF‐8 membranes with <4% extrinsic porosity and a thickness similar to that of commercial polypropylene (pp) separators (23 µm) were fabricated using our approach and tested as molecular sieve components (Figure 4f, Figure S25, Supporting Information).

To investigate the ability of the ZIF‐8 separator to discriminate between Li^+^ on the one hand and S_8_ and LiPSs on the other, electrochemical impedance spectroscopy (EIS) measurements were performed (Figure 4g, Figure S28, Supporting Information). To compare with commercially available pp separators, a set of control measurements was performed as well. The near‐identical impedance (Figure 4g, Figure S28, Supporting Information) of the cells with ZIF‐8 and commercial separators indicates that the MOF membrane thickness and pore size do not inhibit Li^+^ transfer, in agreement with previous reports.^[^ ^29^ ^]^ Additionally, cyclic voltammetry measurements (Figure S27, Supporting Information) suggest that ZIF‐8 does not affect the redox reaction in the model Li‐S battery in the voltage window of 1.5–2.8 V versus Li/Li^+^. The slight variation of the initial impedance between the ZIF‐8 and control cells (Figure 4g, first and second row on the left) might be attributed to the incomplete wetting of the MOF membrane during the fabrication of the cell.^[^ ^12^, ^30^ ^]^ The EIS data for the ZIF‐8 cell (Figure 4g, top‐right) reveal that the MOF membrane successfully blocks the migration of LiPSs, thus preventing the shuttle effect. In contrast, the control cells showed a large impedance variation within the first five cycles (Figure 4g, bottom‐right). Furthermore, the EIS data of the control cells show two semicircles in the high‐ and medium‐frequency regions, as well as an increment of both semicircles from the 1^st^ to the 5^th^ cycle (Figure 4g, bottom‐right). These observations are attributed to the undesirable deposition of Li_2_S/Li_2_S_2_ on the anode and the growth of an additional solid‐electrolyte interface, respectively, and indicate a severe shuttle effect.

To further evaluate the performance of self‐supporting ZIF‐8 membranes obtained through FNN conversion, control cells were constructed with glass‐fiber or commercial pp separators coated with ZIF‐8 through a solvothermal protocol (Figure 4h, Figure S26, Supporting Information). The cells with self‐supporting ZIF‐8 membranes (Figure 4h, violet circles) delivered a high initial capacity of 1276 mAhg^−1^ at a discharge rate of 0.4 C and exhibited an improved cyclability with a small capacity fading rate of 0.12% per cycle and high Coulombic efficiency of above 96.5% for over 200 cycles. In contrast, the cells using the separators coated solvothermally with ZIF‐8 (Figure 4h, red triangles and blue cubes) suffered from a drastic capacity degradation within only a few cycles. In addition, the cells using the self‐standing ZIF‐8 membranes were further tested for 200 cycles at a doubled discharge rate of 0.8 C (i.e., a doubled current density). This higher rate resulted in a reduced initial capacity of 829 mAh g^−1^ and a retention of 62.1% (Figure S33, Supporting Information), in line with the literature.^[^ ^31^ ^]^ This performance demonstrates an interesting cycling performance of our self‐standing ZIF‐8 membranes also at higher current density. Notably, our self‐standing ZIF‐8 separators showed among the best sets of properties when compared to other MOF‐based separators^[32,33]^ (Table S2, Supporting Information). The densified monolithic morphology and crystallinity of the self‐standing ZIF‐8 separators are well‐preserved after 200 charging/discharging cycles at 0.4 C (Figure S34, Supporting Information), indicating mechanical and chemical stability over the battery test.

## 5 Conclusion

We presented a flexible and scalable strategy for the fabrication of MOF films and self‐supporting membranes with tunable hierarchical porosity via vapor phase conversion of metal oxide nanoparticle networks. With this protocol we fabricated pure MOF films with tunable extrinsic interparticle porosity, and controllable thickness in the 80 nm to 230 µm range for areas >100 cm^2^. We determined that for thicknesses in the 12–230 µm range, self‐supporting membranes can be prepared. A 23 µm thick ZIF‐8 self‐standing membrane fabricated via this approach was used as battery separator, and it acted as an effective perm‐selective barrier capable of blocking the migration of polysulfide species while permitting Li^+^ transport in Li‐S batteries, thus resulting in a significantly improved battery cyclability.

## 6 Experimental Section

All chemicals were used without further purification unless otherwise noted. M‐xylene (99.9%), 2‐methylimidazole (99%), trimesic acid (95%), terephthalic acid (98%), and zinc acetate dihydrate (>98%) were purchased from Sigma‐Aldrich. Zinc‐naphthenate (Zn 10%), copper‐naphthenate (Cu 8%), and iron‐naphthenate (Fe 6%) were purchased from Strem. Lithium foil (99.9%), SuperP carbon black, and polyvinylidene fluoride (PVDF) binder (99.5%) were purchased from MTI Corporation. Sulfur (99.98%), 1‐methyl‐2‐pyrrolidinone (NMP) solvent (anhydrous 99.5%), 1,3‐dioxolane (DOL 99.5%), 1,2‐dimethoxyethane (DME 99.5%), and lithium bis(trifluoromethanesulfonyl)imide (LiTFSI, 99.9%) were purchased from Sigma‐Aldrich. Lithium nitrate (LiNO_3_ anhydrous 99.98%) was purchased from Alfa Aesar.

A flame spray pyrolysis (FSP) system (see Figure S29, Supporting Information) with ceramic precursors was used for the preparation of the ZnO FNNs. Liquid precursor solutions of zinc‐naphthenate in m‐xylene with a total metal ion concentration of 0.1, 0.2, and 0.3 mol L^−1^ were supplied to the FSP nozzle and combusted to produce ZnO nanoparticle‐loaded aerosols with customized particle size distributions, as previously discussed.^[^ ^23^ ^]^ Crystalline ZnO FNNs with tunable porosity were synthesized by the self‐assembly of the ZnO aerosols on a variety of substrates placed at a specific height above the burner (HAB). Increasing the HAB from 6 to 20 cm, the nanoparticle network porosity increased from 11% to 98%, respectively. The thickness of the resulting nanoparticle network was linearly controlled by the exposure time of the substrate to the nanoparticle aerosols.

ZnO FNNs were deposited at an HAB of 45 cm on circular glass fiber filters of 15 cm in diameter with the help of a vacuum pump. The coated glass fiber filters were placed in a squared stainless‐steel reaction chamber (16.5 × 16.5 × 2 cm of internal space, wall thickness: 1 cm, with a stainless steel cover lid of 20 × 20 × 1 cm), with the ZnO FNNs facing up (see Figure S30, Supporting Information). The chamber was pre‐filled with 15 g of 2‐MIM placed at the bottom of the internal space, leaving the middle area empty. The membrane was placed over a wire net (15 × 15 cm), in order to leave about 1 cm of space between the membrane and the bottom of the chamber, avoiding direct contact with the ligand powder. The chamber was sealed with a little amount of vacuum grease, to avoid leakage of ligand vapors, then placed in an oven at 120 °C for 18 h. After the reaction time the chamber was left to cool at room temperature naturally before opening.

For the synthesis of ZIF‐8 monoliths, first, a glass vial containing 2‐methylimidazole (2‐MIM) solid crystals was placed into a Teflon lined stainless steel autoclave vial (see Figure S31, Supporting Information) as the vapor source. Thereafter, the ZnO FNNs were placed on a sample holder in the same autoclave. The autoclave was sealed and heated up to varied temperatures (90–150 °C) at a rate of 10 °C min^−1^ using a gravity convection oven and kept for 18 h.

For the synthesis of reference ZIF‐8 particles, zinc acetate dihydrate and 2‐methylimidazole (2‐MIM) were used without further purification in the experiments. The synthesis was performed with an excess amount of 2‐MIM ligand and a minimal amount of water (molar ratio of Zinc acetate dihydrate:2‐MIM:H_2_O = 1:8:600); 1 mmol of zinc acetate dihydrate and 8 mmol of 2‐MIM were dissolved separately in 300 mmol of water. The zinc acetate solution was added to the 2‐MIM solution while being stirred with a magnetic stirrer for 2 h at room temperature. White powders were collected by centrifugation for 10 min, washing twice with ethanol and drying overnight in the oven at 60 °C.

Synchrotron SAXS experiments were conducted in a capillary at the AustroSAXS beamline of Elettra Synchrotron (Trieste, IT). The starting temperature was 25 °C, and the heating rate was 10 °C min^−1^, and the temperature reached 120 °C in approximately 9.5 min.

All XRD patterns were collected using a Bruker system (XRD, D2 Phaser, USA) equipped with Cu K*α* radiation (1.54059 Å).

N_2_ adsorption isotherms of the degassed specimens were recorded at liquid nitrogen temperature using a Tristar II (Micromeritics, USA). The Brunauer‐Emmett‐Teller model was applied to determine the specific surface area in the pressure range of *p*/*p*_0_ between 0.001 and 0.05. The converted ZIF‐8 MOF films were activated by heat treatment at 150 °C for 2 h prior to the tests.

FTIR spectra were recorded utilizing Bruker ALPHA II FTIR Spectrometer with the PLATINUM ATR module from 4000 to 400 cm^−1^.

All SEM images were collected using a Zeiss Ultraplus (FESEM) at 3 kV. All samples were coated with *≈*2 nm Pt before the analysis. HRTEM and HAABF‐STEM were carried out using a JEOL 2100F FEGTEM at 200 kV.

Model of cathode material for Li−S battery were prepared as follows. Elemental sulfur was first mixed with SuperP carbon black powders. The mixture was ground in a corundum mortar for 20 min followed by heating at 155 °C for 2 h in a sealed glass vial. After cooling to room temperature, it was mixed with PVDF in NMP solvent to make a slurry, where the ratio of sulfur, SuperP, and PVDF was 70:20:10 by weight. The slurry was stirred overnight and cast by doctor blading on aluminum (Al) foil current collectors (MTI Corp.). These cathodes were dried in a vacuum oven at 60 °C overnight. Battery assembly: 2032‐type coin cells were assembled in an argon‐filled glovebox. The prepared cathodes were punched into small discs with a diameter of 15 mm. A typical areal mass loading of the active material in cathode was *≈*2.5 mg cm^−2^. Lithium metal foils (diameter/thickness: 16 mm/0.6 mm) were used as the anode. Celgard 2400 (pore size: 0.043 µm) was used as a separator. The electrolyte contained 1.0 M LiTFSI and 0.1 M LiNO_3_ in a mixture of DME and DOL (1:1, vol./vol.). The galvanostatic discharge/charge measurements were conducted on a NEWARE BTS3000 battery test system. The cyclic voltammogram and EIS were carried out using a BioLogic VMP3 multi‐channel potentiostat workstation. All electrochemical measurements were carried out at 20 °C.

Fractal analysis has been carried out via an image‐processing software named FracLac^[^ ^34^ ^]^ following the same procedure previously reported.^[^ ^19^, ^35^ ^]^ In brief, the fractal dimension is evaluated by the slope of a log(*N*) versus log(*ε*), where *N* is the number of pixels of sticks (foreground pixels) and *ε* is the scaling factor. While the lacunarity, a fractal parameter which takes into account the heterogeneity of the system, is calculated via a series of grids with a decreasing size of the box, *ε*, and counting the foreground pixels. For each grid of dimension *ε*, the standard deviation, *σ*, and the mean, µ, of the pixel per box are evaluated. The lacunarity for each grid is then computed as *λ* = (*σ*/µ)^2^ and the average lacunarity is then given by:

(1) $$\begin{array}{c} \bar{\Lambda }=\frac{1}{n}\;\sum_{i}\left(\frac{\sigma_{i}}{\mu_{i}}\right)+1 \end{array}$$

where the summation is over all the grids with dimension *ε*. Note that this equation has a “+1” added for completeness to take into account the eventuality of a total homogeneous (no variation in the pixel distribution) image.

## Conflict of Interest

The authors declare no conflict of interest.

## Supporting information

Supporting Information
