# Supplementary material for: Hierarchical Metal‐Organic Framework Films with Controllable Meso/Macroporosity
Source: Adv Sci (Weinh). 2020 Nov 13;7(24):2002368. doi: 10.1002/advs.202002368 (PMC7740079; doi:10.1002/advs.202002368)
Supplement: Supplementary file 1 — Supporting Information [file ADVS-7-2002368-s001.pdf]

© 2020 Wiley-VCH GmbH

ADVANCED SCIENCE
Open Access

Supporting Information

for *Adv. Sci.,* DOI: 10.1002/advs.202002368

Hierarchical Metal-Organic Framework Films with Controllable Meso/Macroporosity

Renheng Bo, Mahdiar Taheri, Borui Liu, Raffaele Ricco, Hongjun Chen, Heinz Amenitsch, Zelio Fusco, Takuya Tsuzuki, Guihua Yu, Rob Ameloot, Paolo Falcaro, and Antonio Tricoli**

Supporting Information

Three-Dimensional Structuring of Hierarchical Metal-Organic Frameworks Monoliths

Renheng Bo[1,#], Mahdiar Taheri[2,#], Borui Liu[1,#], Raffaele Ricco[3], Hongjun Chen[1], Heinz Amenitsch[4], Zelio Fusco[1], Takuya Tsuzuki[2], Guihua Yu[5], Rob Ameloot[6], Paolo Falcaro[3,] and Antonio Tricoli[1,*]*

Contents

Figure S1 Engineering of the precursor ceramic particle size. 4

Figure S2 *In situ* synchrotron SAXS and WAXS....6

Figure S3 Engineering of the extrinsic porosity of the fractal precursor ZnO nanoparticle networks (FNNs)....7

Figure S4 XRD patterns of the 90% porous ZnO FNN before and after conversion at 150 and 180 °C 8

Figure S5 Optical image of the 90% porous ZnO FNN converted to ZIF-8 at 180 °C. 9

Figure S6 XRD patterns of the 97% porous ZnO FNN before and after conversion at 150 °C....10

Figure S7 XRD patterns of the 98% porous ZnO FNN before and after conversion at 150 °C....11

Figure S8 Morphological studies of the 98% porous ZnO FNN with a thickness of 7 μm converted into ZIF-8 at 150 °C. 12

Figure S9 Morphological studies of the 98% porous ZnO FNN with a thickness of 33 μm converted into ZIF-8 at 150 °C. 13

Figure S10 XRD and FTIR characterizations of the ZnO nanoparticle network converted into ZIF-8 as a function of the conversion temperature. 14

Figure S11 Top view SEM images of ZIF-8 films converted at varied temperatures. 16

Figure S12 The lacunarity evaluated by the pixel distribution of the images 17

Figure S13 Fractal dimension....18

Figure S14 Engineering of the extrinsic porosity and hierarchy. 19

Figure S15 Top view SEM images of ZIF-8 monolith with 66 ± 2% extrinsic porosity. 20

Figure S16 Top view SEM images of ZIF-8 monolith with 43 ± 2% extrinsic porosity. 21

Figure S17 Top view SEM images of ZIF-8 monolith with 27 ± 2% extrinsic porosity. 22

Figure S18 Top view SEM images of ZIF-8 monolith with 11 ± 2% extrinsic porosity. 23

Figure S19 Top view SEM images of ZIF-8 monolith with 4 ± 2% extrinsic porosity. 24

Figure S20 SEM images of 80 and 160 nm thick ZIF-8 films obtained by converting FNN....25

Figure S21 TGA characterization of the 230 μm thick ZIF-8 monolith. 26

Figure S22 XRD analysis on the self-standing ZIF-8 monolith (*ca* 230 μm thick) before and after conversion. 27

Figure S23 Optical images of *ca* 0.66 mm thick ZnO FNNs with different designs....28

Figure S24 Schematic of the approach for fabrication of self-standing MOF monoliths, and SEM images of a representative 12 μm thick self-standing ZIF-8 monolith....29

Figure S25 SEM images of the precursor ZnO FNN and the converted ZIF-8 membrane. 31

Li-S system....32

Further characterizations of the batteries 32

Figure S26 Comparison of Coulombic efficiencies of the batteries with the monolithic MOF membrane and other separators. 33

Figure S27 Cyclic voltammogram of a control cell with only ZIF-8 monolith as a cathode and Al foil as the current collector for the first 10 cycles....34

Figure S28 EIS plots of the control Li–S battery. 35

Figure S29 Schematic of the flame spray pyrolysis system used for the synthesis of the precursor ceramic nanoparticles and their self-assembly into a nanoparticle network (FNN)..............................36

Figure S30 Optical images of a customized conversion reactor...37

Figure S31 Teflon lined stainless steel autoclave vial..38

Calculations..39

Table S1 Table of extrinsic porosity calculations and N_2 adsorption analysis. 41

Table S2 Performance comparison of Li–S batteries with MOF-based separators. 42

The effect of pressure on the ZIF-8 film..44

Figure S32 XRD of partially converted FNN precursor at 150 °C under an unsaturated pressure of 1070.90 pa. 45

Figure S33 Cycling performance of self-standing ZIF-8 separator at a discharge rate of 0.8 C. 46

Figure S34 Post battery characterizations. 47

References 48

Engineering the size of the ceramic nanoparticle precursor was achieved by controlling the metal precursor concentration supplied to the flame spray pyrolysis burner. The injection of a solution with lower concentrations of the metal precursor leads to aerosols composed by particles with smaller size. Here, the metal precursor concentration was varied from 0.1 to 0.3 mol L^{-1}.Transmission electron microscopy (TEM) analysis was carried out by collection of the ZnO nanoparticles from the obtained nanoparticle networks (Figure S1a-c). Representative particle size distributions (Figure S1d) were calculated by counting 300 ZnO nanoparticles for all different precursor concentrations showing a good control over the particle size of the ceramic precursor. This control over size is crucial to achieve full conversion from ZnO to ZIF-8 by gas phase; indeed, this reaction is generally limited to a thin layer of 10-15 nanometres from the surface of the ceramic particles.[1, 2] A metal precursor concentration of 0.3 mol L^{-1} was selected as it afforded: 1) particles smaller than 20 nm (whereas the limit for gas phase conversion is *ca* 15 nm),[1, 2] and 2) the highest concentration of nanoparticle in the aerosol. Thus the nanoparticle network growth rate was optimized.

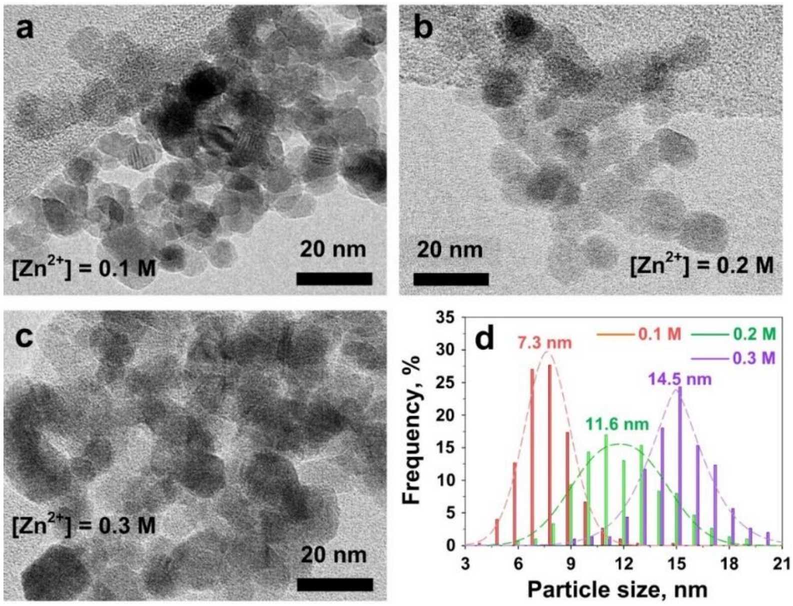

Figure S1 Engineering of the precursor ceramic particle size. a-c, Representative TEM images of engineered ZnO nanoparticles made by flame spray pyrolysis with liquid precursor concentrations of 0.1, 0.2 and 0.3 mol.L^{-1}. **d,** Particle size distributions of the flame-made precursor ZnO nanoparticles with liquid precursor concentrations of 0.1, 0.2 and 0.3 mol.L^{-1}.

The gas-phase conversion kinetics of the engineered flame-made ZnO nanoparticles, obtained with a liquid precursor concentration of 0.3 mol.L^{-1} (*vide supra*), to ZIF-8 was monitored via *in situ* synchrotron small-angle X-ray scattering (SAXS). Figure S2 shows the first 18 minutes of reaction of ZnO with 2-MIM vapors from room temperature up to maximum 120 °C (whereas the melting point of 2-MIM is *ca* 142 °C), for both flame-made ZnO nanoparticles collected from the ZnO nanoparticle networks and for commercial ZnO particles. The appearance of characteristic peaks of ZIF-8 lattice plane (001) in SAXS (Figure S2a, left), and lattice plane (112), (022) as well as (013) in wide-angle X-ray scattering (WAXS) (Figure S2a, right) indicates that the flame-made ZnO nanoparticles (collected from the FNNs) react with 2-MIM to produce ZIF-8 at *ca* 75 °C after *ca* 5 min, while for the commercial ZnO powder the ZIF-8 characteristic peaks started being observed only at *ca* 12.5 min after reaching the maximum temperature (Figure S2b). The peaks observed at the beginning of the reaction at q = 10 and 12 1/nm correspond to those of the crystalline 2-MIM powder ligand. These findings reveal that the engineered flame-made ZnO nanoparticles are highly reactive precursors for MOF conversion.

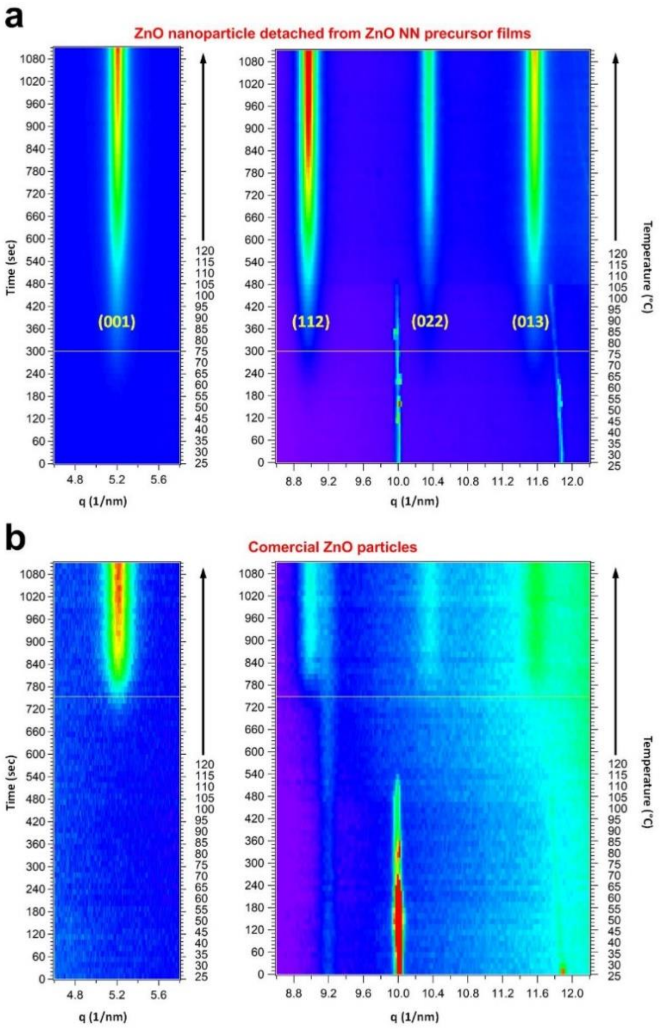

Figure S2 ***In situ*** **synchrotron SAXS and WAXS monitoring of the reaction kinetics of the flame-made ZnO nanoparticles with 2-MIM vapours.** *In situ* monitoring of the conversion of (**a**) flame-made ZnO nanoparticles detached from ZnO FNN precursor films and (**b**) commercial ZnO particles.

In addition to the particle size, engineering the extrinsic porosity of the fractal nanoparticle networks (FNNs) is the key for successful conversion of the ceramic precursor particles into a monolithic MOF film. This is particularly important as during ceramic to MOF conversion the solid fraction volume of the film increases significantly (e.g. 17 times from ZnO to ZIF-8)[2], thus, a sufficient extrinsic porosity of the precursor ceramic film is required to avoid strain and ensure continuous exposure of the ceramic precursor to the ligand vapours. To investigate the effect of the FNN extrinsic porosity on the converted MOF, ZnO films with porosity ranging from 11% to 98%, here named extrinsic porosity, were fabricated by changing the height above the flams spray pyrolysis burner (HAB) at which the substrates were placed, as described elsewhere.[3]

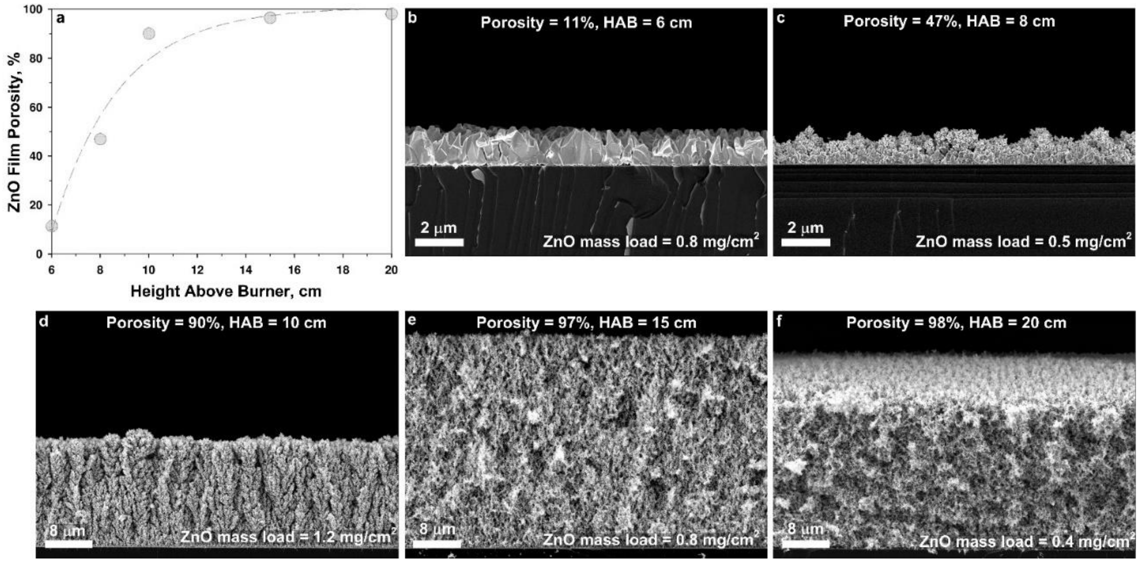

Figure S3 Engineering of the extrinsic porosity of the fractal precursor ZnO nanoparticle networks (FNNs). a, Extrinsic porosity of ZnO FNNs as a function of the height above the burner (HAB). **b-f,** Cross-sectional SEM images of ZnO FNNs with engineered extrinsic porosities from 11 - 98% and various ZnO mass loading. It should be noted that the thickness of the 97% porous FNN is higher than that of the 98% one, due to the doubled mass loading comparing to the former. All samples were coated with a thin layer of Pt (*ca* 2 nm) to facilitate imaging.

Characterization of the converted ZnO FNNs (90% extrinsic porosity) into ZIF-8 films. XRD measurements were performed on the ZnO FNN before and after reaction with the 2-MIM vapour at 150 °C and 180 °C. While some of the ZIF-8 characteristic peaks were detected at 150 °C, the reaction was still not completed upon 18 h at this temperature. Further increasing the temperature to 180 °C and reacting for 18 h resulted in disappearance of the ZnO characteristic peaks and in rise of the ZIF-8 characteristic reflections. However, cracks were observed as shown in Figure S5.

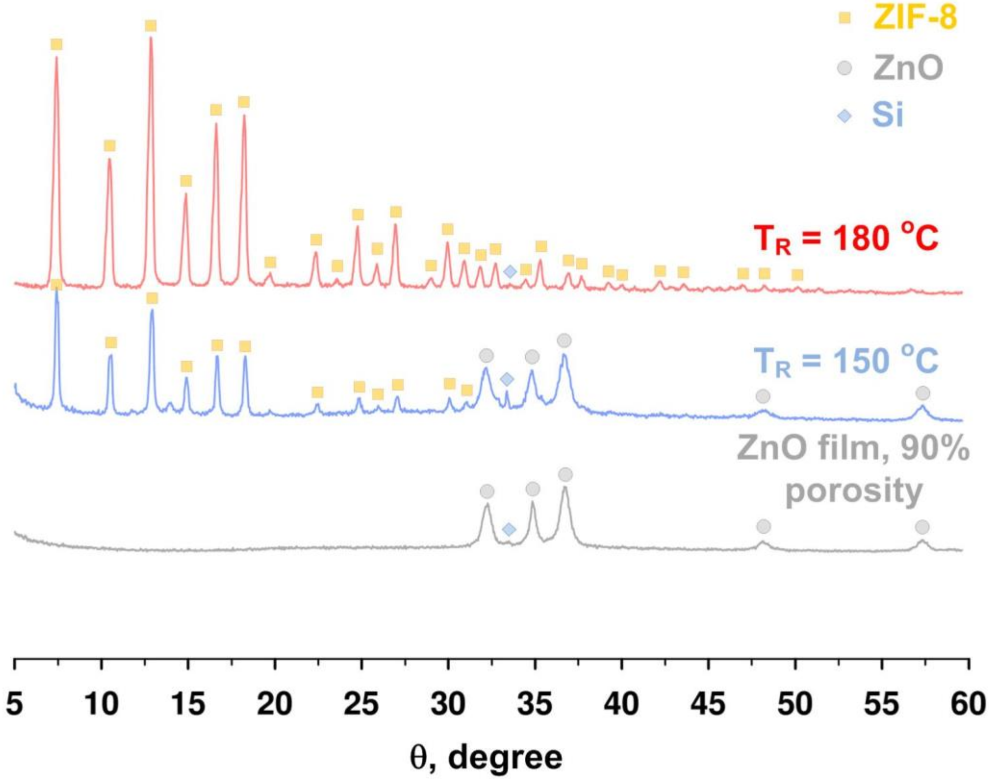

Figure S4 XRD patterns of the 90% porous ZnO FNN before and after exposure to 2-MIM vapour at 150 and 180 °C. XRD patterns of ZnO FNN with 90% extrinsic porosity before (grey line) and after conversion at 150 °C (light blue line) and 180 °C (red line).

Optical image of the 90% porous ZnO FNN converted to ZIF-8 at 180 °C. Upon the reaction, formation of cracks and partial delamination of the resulting film is observed. It can be attributed to the insufficient void space that cannot accommodate the 17-fold expansion of the crystal lattice from ZnO to ZIF-8.[2] This experimental observation is in line with our calculation (see Supplementary Calculation Equation 1) that indicates a minimum required extrinsic porosity of 94.12% for full conversion of ZnO nanoparticle network to ZIF-8 monoliths.

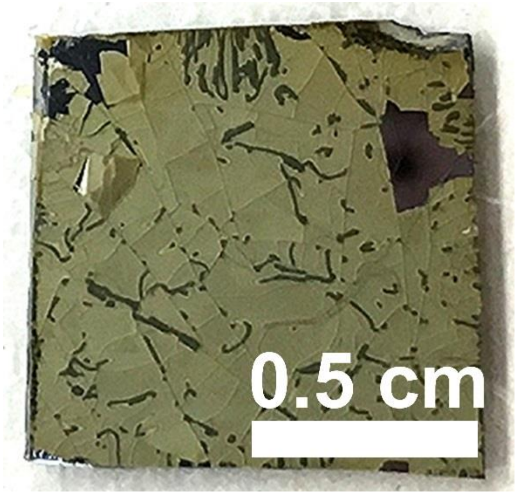

Figure S5 Optical image of the 90% porous ZnO FNN converted to ZIF-8 at 180 °C. The formation of cracks and partial delamination of the resulting film is observed at this lower porosity.

XRD measurements were performed on the ZnO FNN (97% extrinsic porosity) before and after reaction with the 2-MIM vapour at 150 °C for 18 h. The typical diffraction peaks of the wurtzite (ZnO)[4] (Figure S6 grey line) disappeared after reaction with 2-MIM. After the reaction the typical diffraction pattern of ZIF-8 (Figure S6 blue line) was observed.[2]

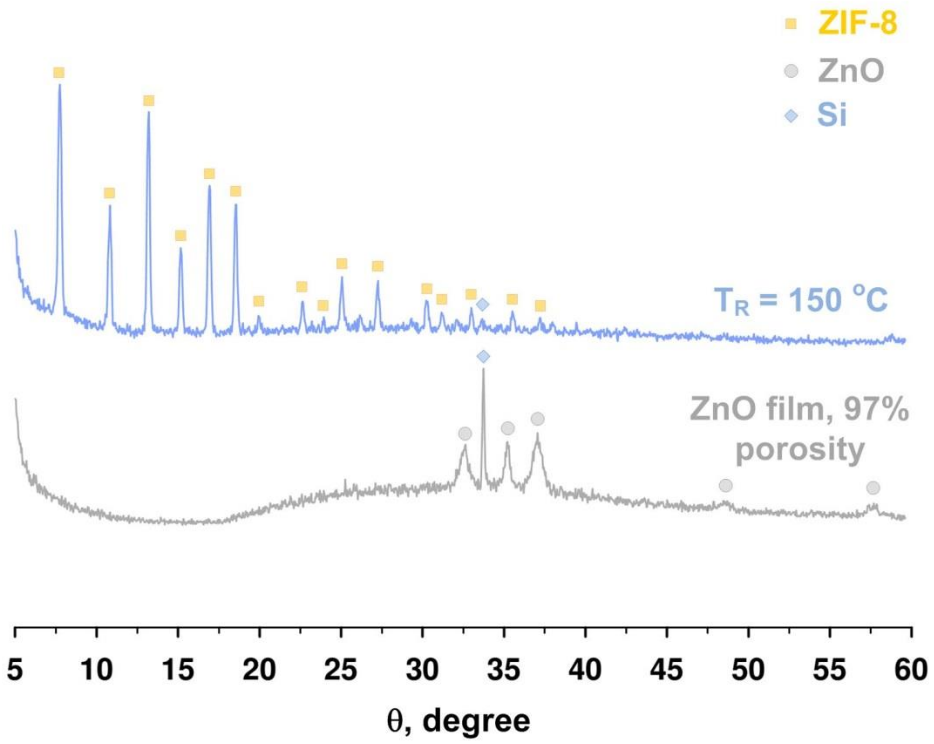

Figure S6 XRD patterns of the 97% porous ZnO FNN before and after exposure to 2-MIM vapour at 150 °C. The XRD diffraction patterns of the 97% ZnO FNN before (grey line) and after conversion at 150 °C (light blue) confirm the complete conversion.

XRD measurements were performed on the ZnO FNN (98% extrinsic porosity) before and after reaction with the 2-MIM vapour at 150 °C for 18 h. The typical diffraction peaks of wurtzite (ZnO)[4] (Figure S7 grey line) disappeared after reaction with 2-MIM. After reaction, the typical diffraction pattern of ZIF-8[2] (Figure S7 blue line) was observed. In comparison to the XRD spectra of the MOFs converted from the 97% porous nanoparticle networks, a sharpening of the diffraction peaks was observed, indicating growth of larger MOF crystals.

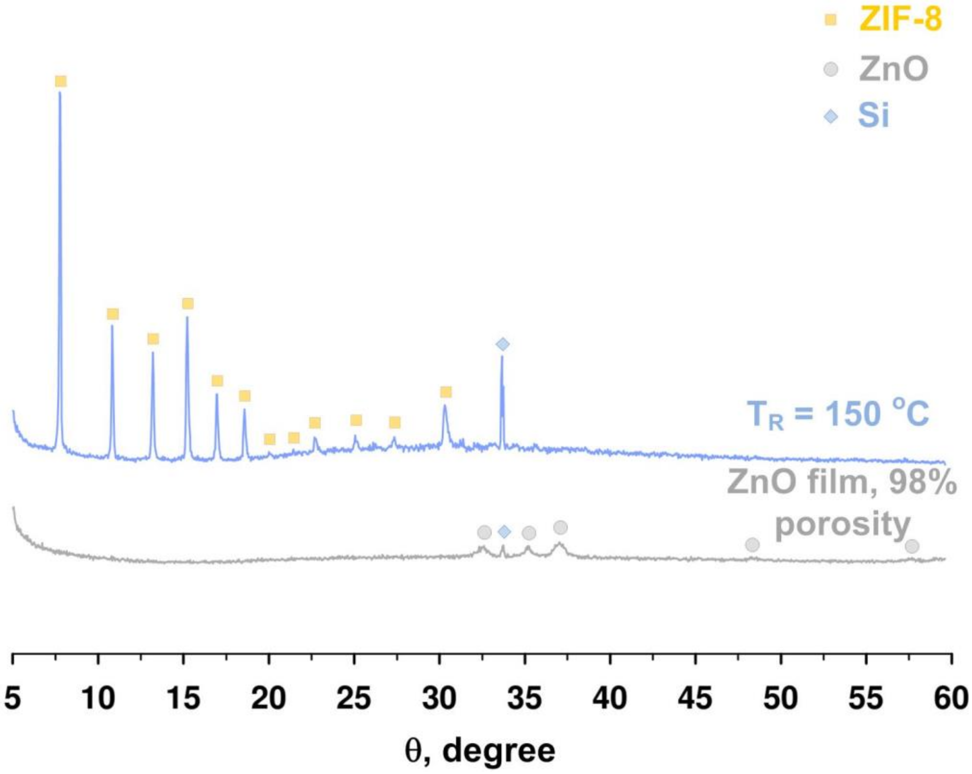

Figure S7 XRD patterns of the 98% porous ZnO FNN before and after exposure to 2-MIM vapour at 150 °C. The XRD diffraction patterns of the 98% ZnO FNN before (grey line) and after conversion at 150 °C (light blue) confirm the complete conversion.

Figure S8 shows the cross-sectional SEM images of a 98% porous ZnO FNN (Figure S8a, b) and the ZIF-8 monolithic film (Figure S8c, d) obtained through reaction with 2-MIM vapour at 150 °C for 18 hours. A film thickness contraction factor of *ca* 2.8 was observed with respect to the precursor film, resulting in a dense ZIF-8 monolithic structure with only *ca* 4 ± 2% extrinsic porosity.

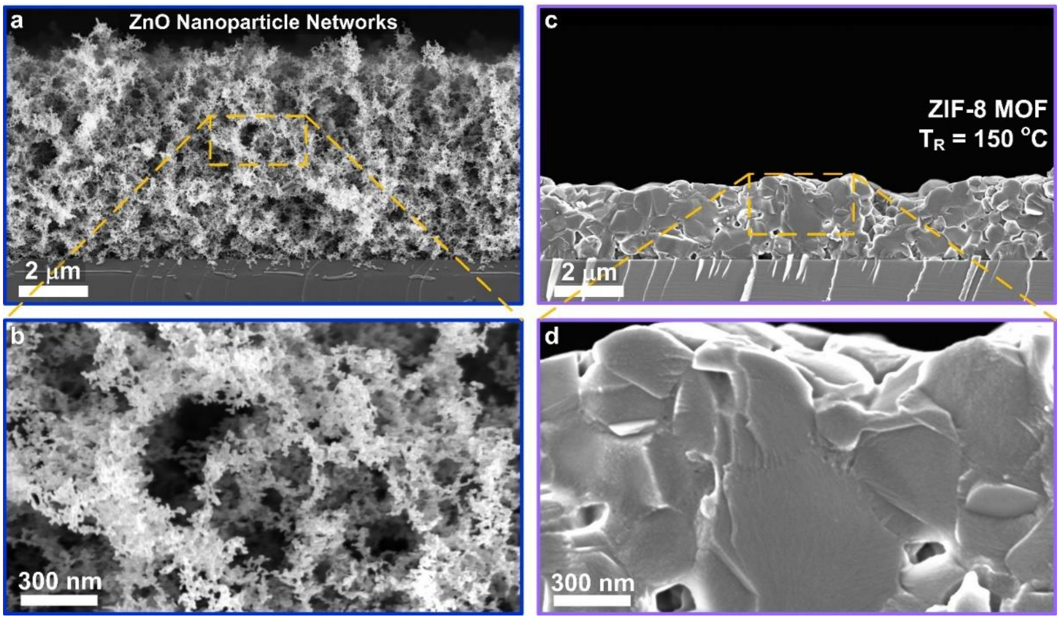

Figure S8 Morphological studies of the 98% porous ZnO FNN with a thickness of 7 μm converted into ZIF-8 at 150 °C. a, Cross-sectional SEM image of a 7 μm thick precursor ZnO FNN and (**b**) the corresponding higher magnification image. **c,** Cross-sectional SEM image of the converted ZIF-8 film and (**d**) the corresponding higher magnification. All samples were coated with a thin layer of Pt (*ca* 2 nm) to avoid charging.

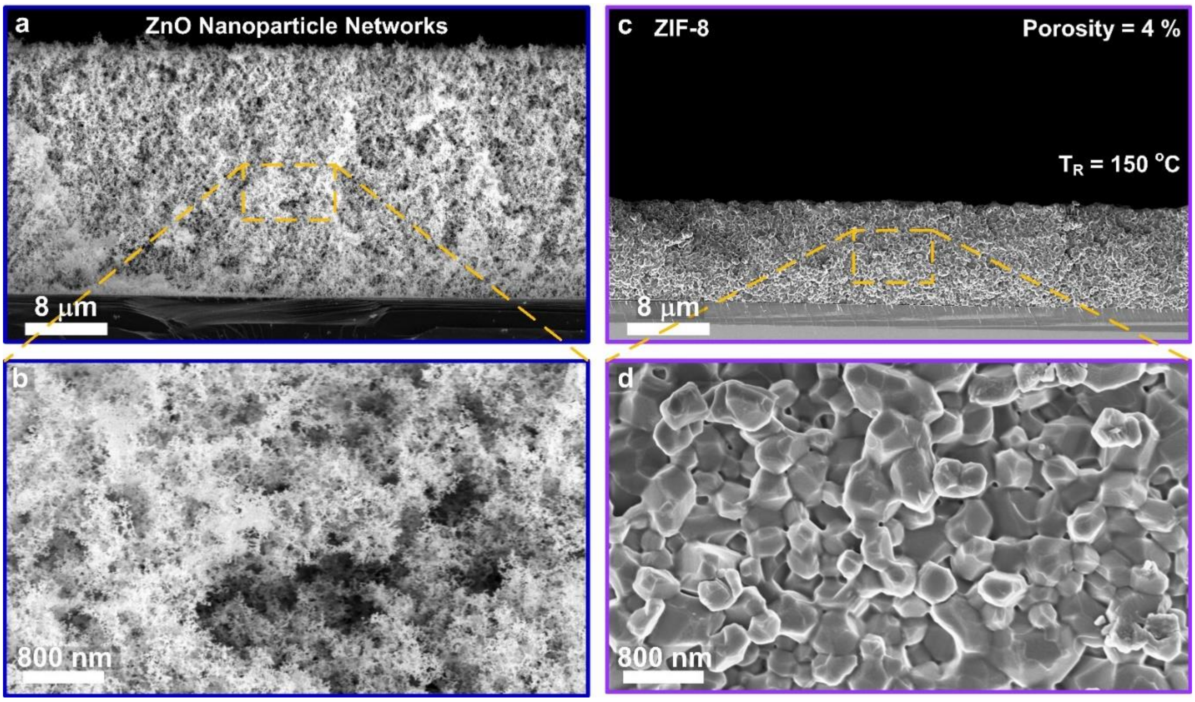

Figure S9 Morphological studies of the 98% porous ZnO FNN with a thickness of 33 μm converted into ZIF-8 at 150 °C. a, Cross-sectional SEM image of a 33 μm thick precursor ZnO FNN and (**b**) the corresponding higher magnification image. **c,** Cross-sectional SEM image of the converted ZIF-8 film and (**d**) the corresponding higher magnification. All samples were coated with a thin layer of Pt (*ca* 2 nm) to avoid charging.

To further verify the full conversion of the ceramic precursor and investigate the impact of reaction temperature on the conversion of the precursor ZnO FNN into ZIF-8 monoliths, a series of XRD and FTIR characterizations were performed and compared to ZIF-8 powders obtained by an established aqueous synthesis.[5] Notably and in line with the *in situ* synchrotron measurements (*vide supra*), all reaction temperatures from 90 to 150 °C resulted in the full conversion of the 98% porous ZnO nanoparticle networks into ZIF-8. The XRD characteristic peaks of wurtzite (ZnO)[4] of the FNNs (Figure S10a, dark blue line) matches well those of the ZnO nanoparticles (Figure 10a, black line). Upon reaction with 2-MIM, the ZnO lattice diffraction peaks were replaced by the characteristic peaks of the ZIF-8 (sodalite)[2] at all tested temperatures (Figure S10a, ZIF-8 on Si wafer at 90 – 150°C) indicating full conversion of the precursor FNNs. Fourier-transform infrared spectroscopy (FTIR) measurements were carried out on the same set of samples. Both the ZnO FNN on Si wafer (Figure S10b, dark blue line) and the pure ZnO nanoparticles (Figure S10, black line) FTIR spectra show the characteristic bond of Zn-O stretching at 440 cm^{-1}. Comparison of the FTIR spectra of the pure ZIF-8 particles made by aqueous synthesis (Figure S10b, dark cyan line) and the ZIF-8 monoliths obtained by conversion of the ZnO FNN at 90 – 150 °C (Figure S10, ZIF-8 films on Si wafer) confirms that pure ZIF-8 films were obtained. The observed characteristic modes at 1500 – 1350, 1350 – 900, 800 – 660 and 423 cm^{-1} are corresponding to imidazole ring stretching, in plane bending of imidazole ring, out of plane bending of imidazole ring and Zn-N stretching, respectively. In the reacted samples, the Zn-O stretching at 440 cm^{-1} of the FNN were completely replaced by the Zn-N stretching at 423 cm^{-1}. To be noted, the broad peaks observed at *ca* 520 – 500 cm^{-1} in the bare Si wafer and in all films on Si wafer are attributed to the Si-O rocking,[6] which is expected to arise from the thin SiO_2 layer on the Si-wafers.

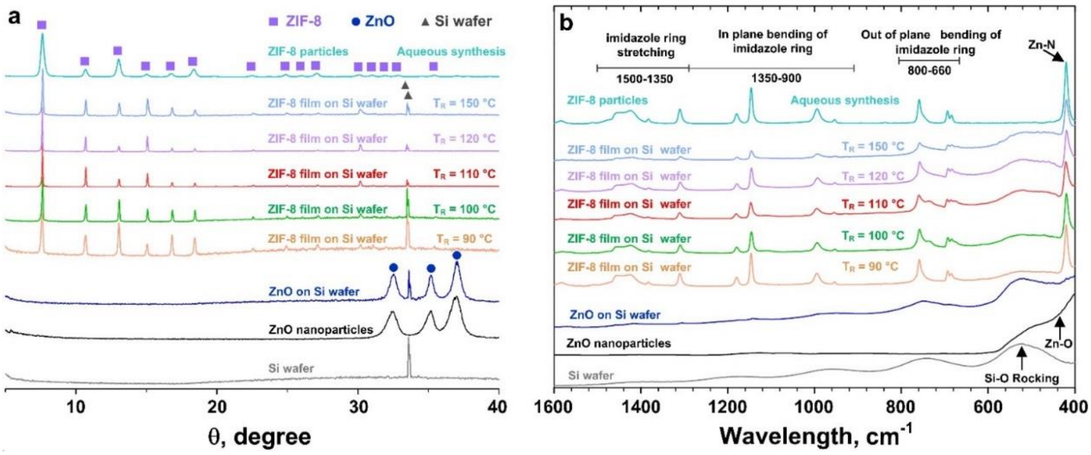

Figure S10 XRD and FTIR characterizations of the ZnO nanoparticle network converted into ZIF-8 as a function of the conversion temperature. a, XRD patterns of a bare Si wafer used as

substrate (grey line), pure flame-made ZnO nanoparticles (black line), ZnO nanoparticles on a Si wafer (dark blue line), the converted ZIF-8 monoliths at different conversion temperatures from 90 to 150 °C, and pure ZIF-8 particles obtained via the aqueous synthesis (dark cyan line). **b,** FTIR spectra of a bare Si wafer used as substrate (grey line), pure flame-made ZnO nanoparticles (black line), ZnO nanoparticles on a Si wafer (dark blue line), the converted ZIF-8 monoliths at different conversion temperatures from 90 to 150 °C, and pure ZIF-8 particles obtained via the aqueous synthesis (dark cyan line).

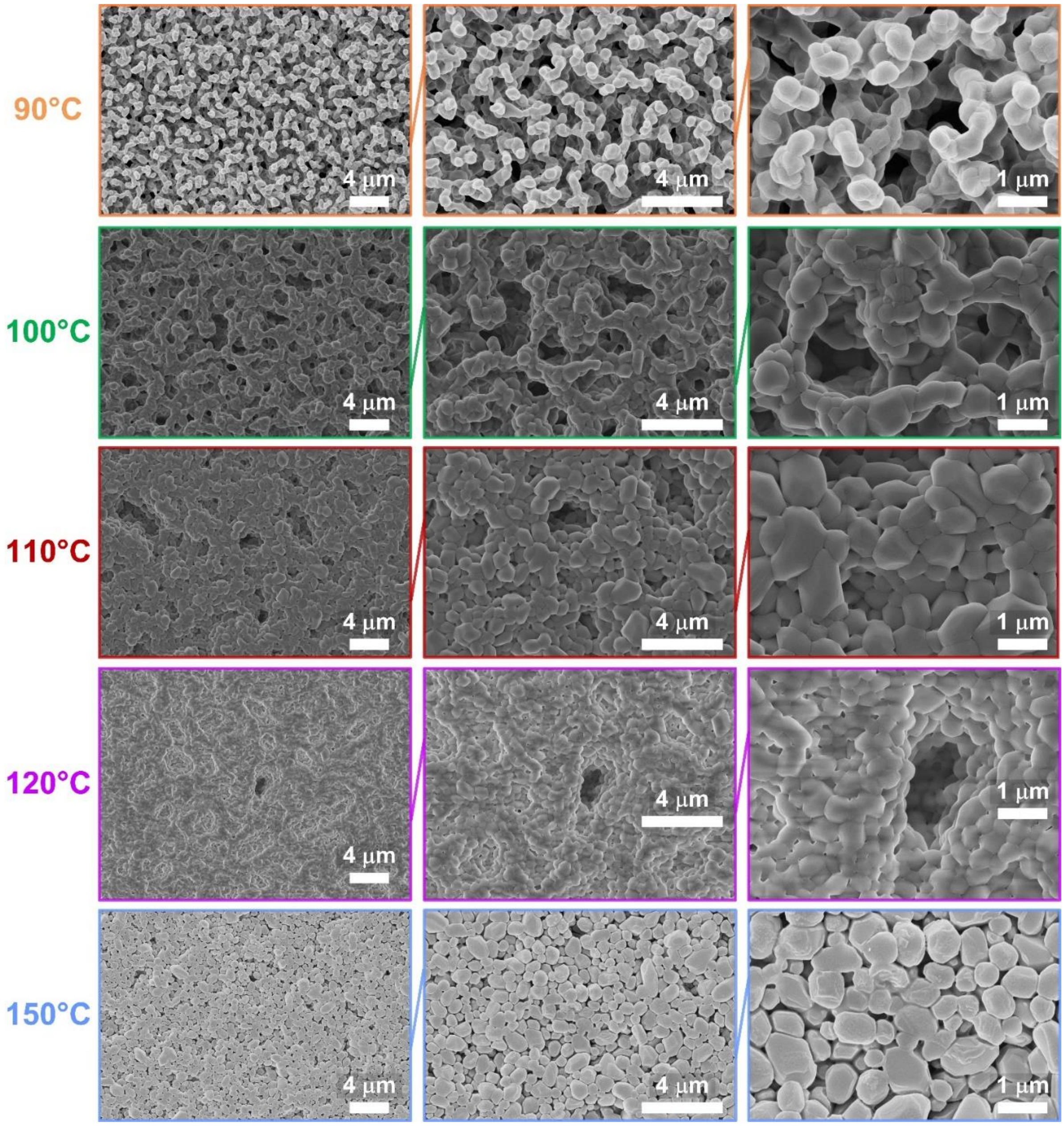

Figure S11 Top view SEM images of ZIF-8 films converted at varied temperatures. Colour codes: 90 °C is light orange, 100 °C is green, 110 °C is red, 120 °C is light purple and 150 °C is light blue.

The lacunarity, a fractal parameter, which takes into account the heterogeneity of the system, is calculated via a series of grids with a decreasing size of the box, ε, and counting the foreground pixels. For each grid of dimension ε, the standard deviation, σ, and the mean, μ, of the pixel per box are evaluated. The lacunarity for each grid is then computed as $\lambda = (\sigma/\mu)^2$ and the average lacunarity is then given by:

$$\overline{\Lambda} = \frac{1}{n}\sum_{i}\left(\frac{\sigma_i}{\mu_i}\right) + 1$$

where the summation is over all the grids with dimension ε. Note that this equation has a '+1' added for completeness to take into account the eventuality of a total homogeneous (no variation in the pixel distribution) image.

The lacunarity of pure ZnO (Figure S12) peaking at 100 – 400 nm is well in line with previous report.[7]

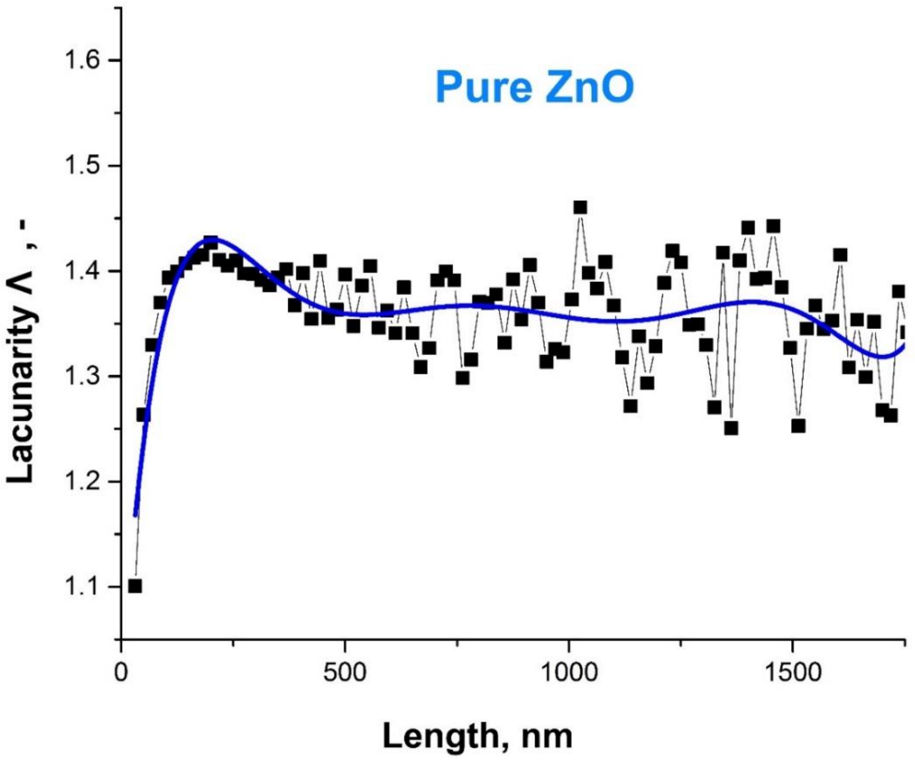

Figure S12 The lacunarity evaluated by the pixel distribution of the images for pure ZnO nanoparticle network.

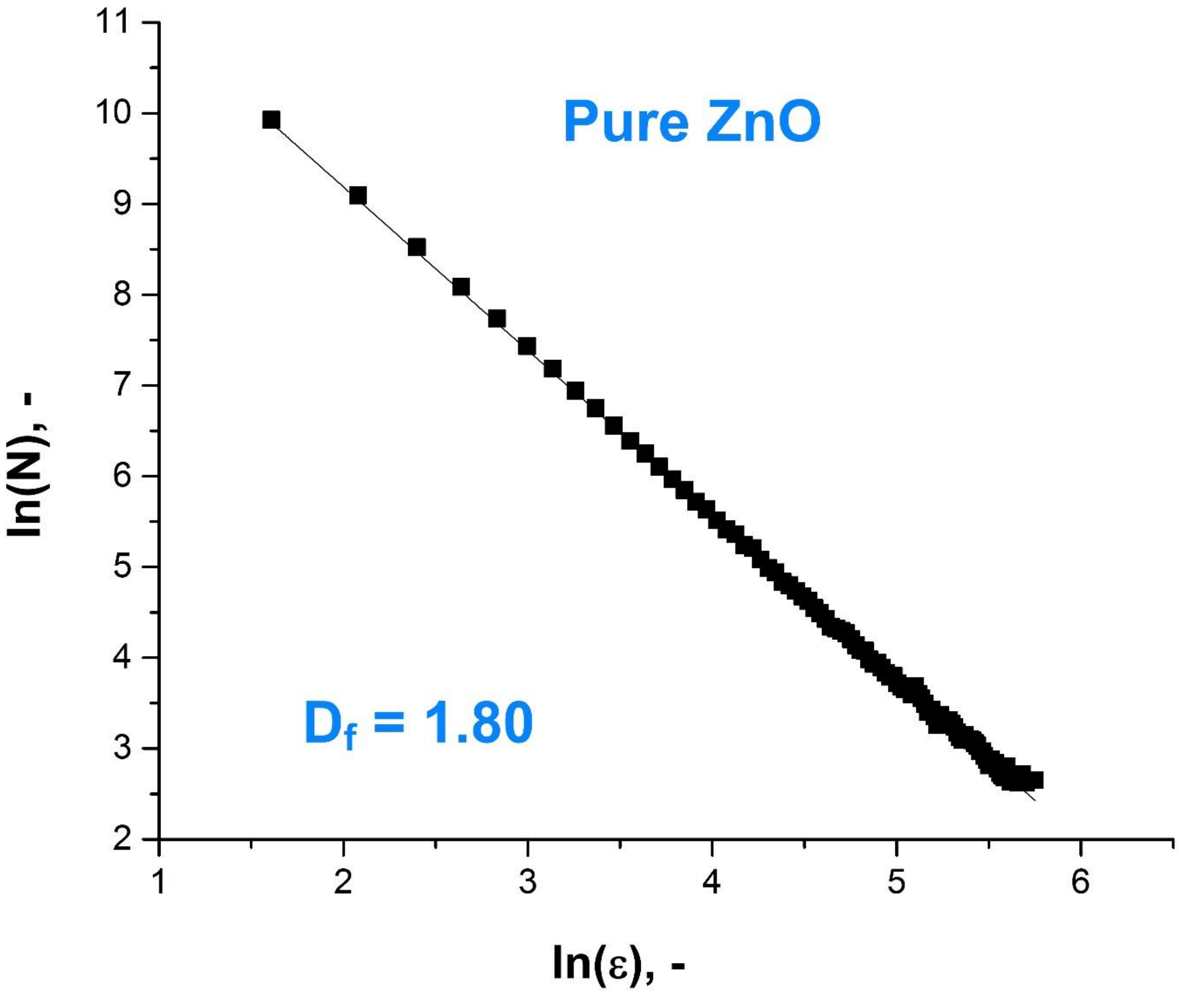

Figure S13 Fractal dimension of pure ZnO nanoparticle network.

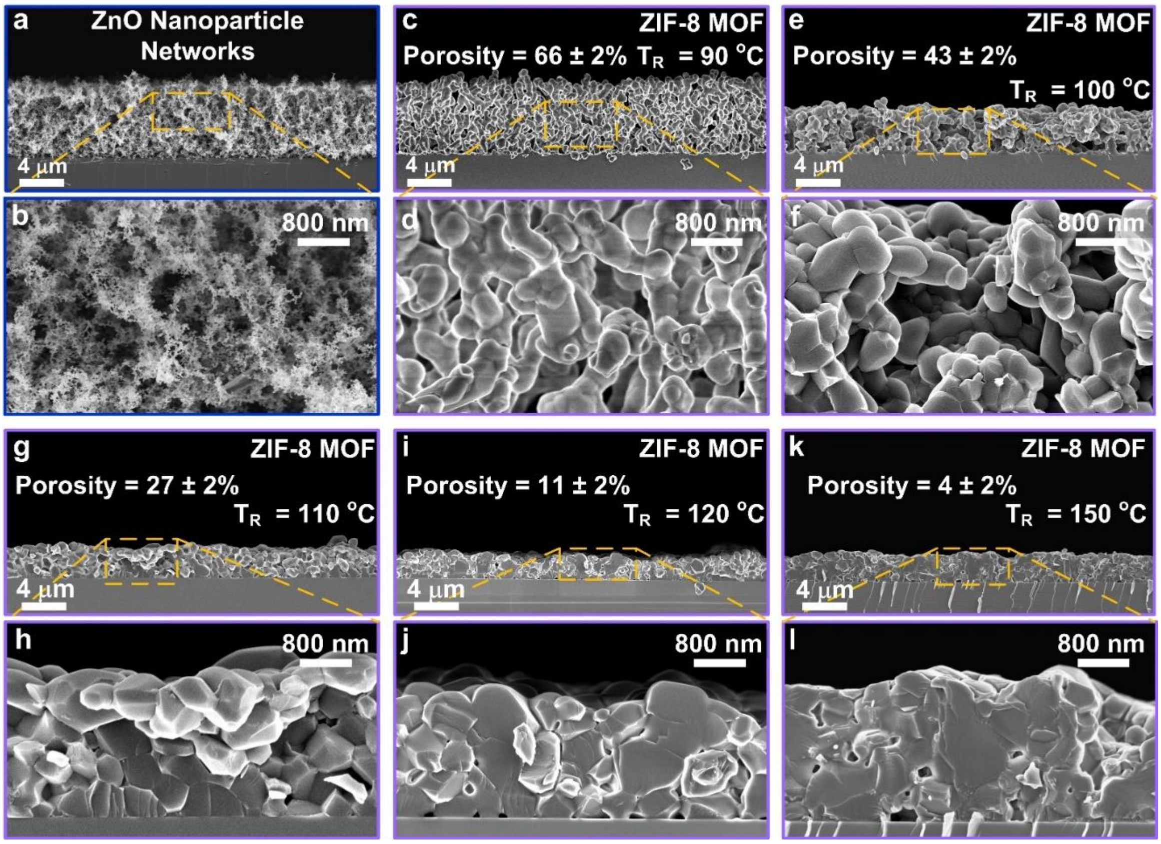

Figure S14 Engineering of the extrinsic porosity and hierarchy. a, Cross-sectional SEM images of a 98% porous ZnO FNN precursor utilized for ZIF-8 monolith fabrication and (**b**) the corresponding high magnification SEM image of **a**. **c,** Cross-sectional SEM images of the ZIF-8 monolithic film with an extrinsic porosity of 66 ± 2% obtained at 90 °C and (**d**) the corresponding high magnification SEM image of **c**. **e,** Cross-sectional SEM images of the ZIF-8 monolithic film with an extrinsic porosity of 43 ± 2% obtained at 100 °C and (**f**) the corresponding high magnification SEM image of **e**. **g,** Cross-sectional SEM images of the ZIF-8 monolithic film an extrinsic porosity of 27 ± 2% obtained at 110 °C and (**h**) the corresponding high magnification SEM image of **g**. **i,** Cross-sectional SEM images of the ZIF-8 monolithic film with an extrinsic porosity of 11 ± 2% obtained at 120 °C and (**j**) the corresponding high magnification SEM image of **i**. **k,** Cross-sectional SEM images of the ZIF-8 monolithic film with an extrinsic porosity of 4 ± 2% obtained at 150 °C and (**l**) the corresponding high magnification SEM image of **k**. All samples were coated with a thin layer of Pt (*ca* 2 nm) to avoid charging.

Representative top view SEM images of a ZIF-8 monolithic film with an extrinsic porosity of 66 ± 2%, which are in line with the cross-sectional SEM images (**Figure 2**, Figure S15) obtained from the same film.

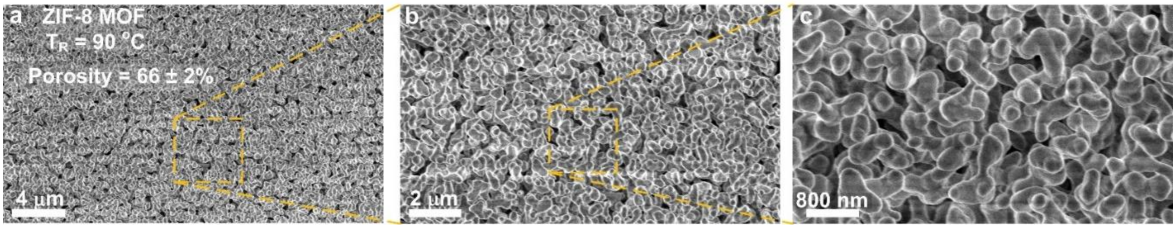

Figure S15 Top view SEM images of ZIF-8 monolith with 66 ± 2% extrinsic porosity. Increased magnifications are presented from **a** to **c**. No coating was deposited prior to SEM analysis.

Representative top view SEM images of a ZIF-8 monolithic film with an extrinsic porosity of 43 ± 2%, which are in line with the cross-sectional SEM images (**Figure 2**, Figure S16) obtained from the same film.

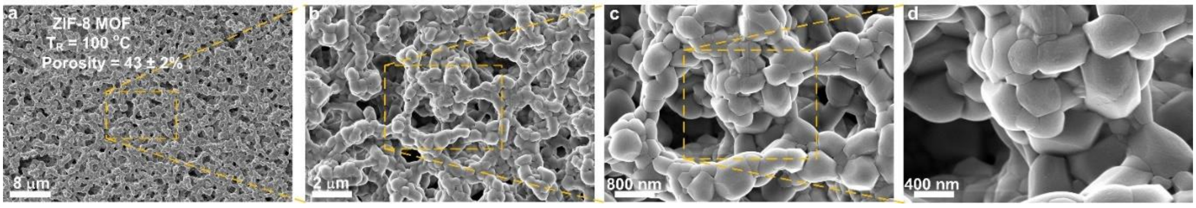

Figure S16 Top view SEM images of ZIF-8 monolith with 43 ± 2% extrinsic porosity. Increased magnifications are presented from **a** to **d**. All samples were coated with a thin layer of Pt (*ca* 2 nm) to avoid charging.

Representative top view SEM images of a ZIF-8 monolithic film with an extrinsic porosity of 27 ± 2%, which are in line with the cross-sectional SEM images (Figure S17) obtained from the same film.

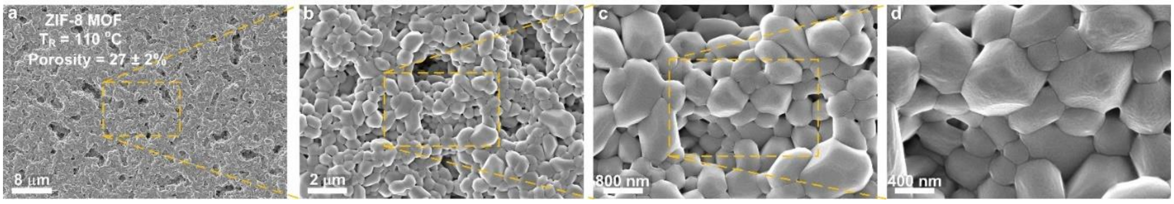

Figure S17 Top view SEM images of ZIF-8 monolith with 27 ± 2% extrinsic porosity. Increased magnifications are presented from **a** to **d**. All samples were coated with a thin layer of Pt (*ca* 2 nm) to avoid charging.

Representative top view SEM images of a ZIF-8 monolithic film with an extrinsic porosity of 11 ± 2%, which are in line with the cross-sectional SEM images (**Figure 2**, Figure S18) obtained from the same film.

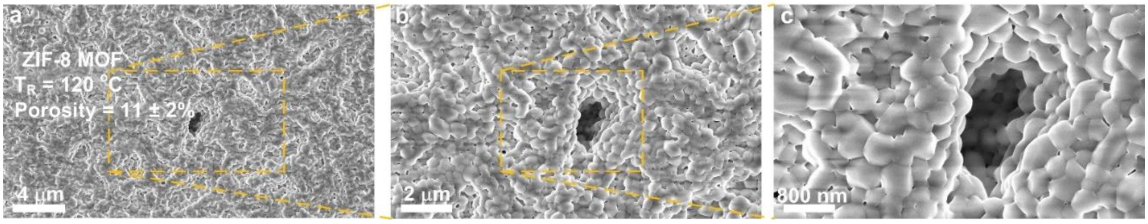

Figure S18 Top view SEM images of ZIF-8 monolith with 11 ± 2% extrinsic porosity. Increased magnifications are presented from **a** to **c**. No coating was deposited prior to SEM analysis.

Representative top view SEM images of a ZIF-8 monolithic film with an extrinsic porosity of 4 ± 2%, which are in line with the cross-sectional SEM images (**Figure 2**, Figure S19) obtained from the same film.

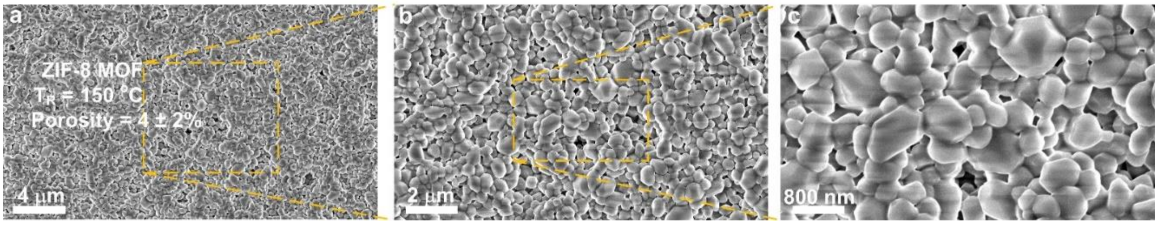

Figure S19 Top view SEM images of ZIF-8 monolith with 4 ± 2% extrinsic porosity. Increased magnifications are presented from **a** to **c**. No coating was deposited prior to SEM analysis.

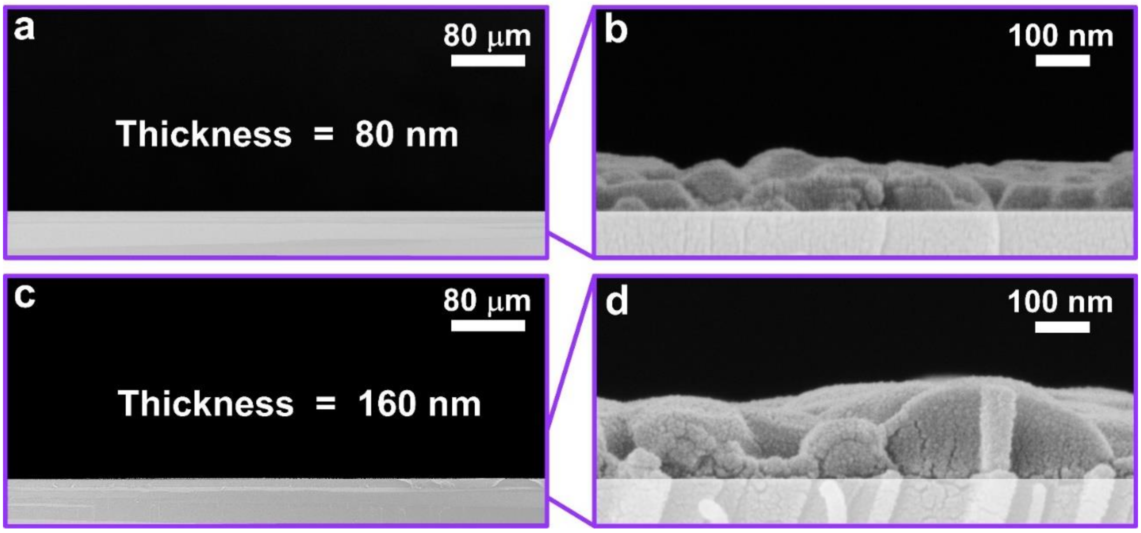

Figure S20 SEM images of 80 and 160 nm thick ZIF-8 films obtained by converting FNN. a, Cross-sectional SEM images of 80 nm thick ZIF-8 film and (**b**) the corresponding high magnification SEM image of **a**. **c,** Cross-sectional SEM images of 160 nm thick ZIF-8 film and (**d**) the corresponding high magnification SEM image of **c**. All samples were coated with a thin layer of Pt (*ca* 2 nm) to avoid charging.

TGA measurements were carried out with the pure flame-made ZnO nanoparticles, 230 μm-thick ZIF-8 monoliths made by conversion of the ZnO FNN, and ZIF-8 particles prepared using the aqueous synthesis method. Both the ZIF-8 monoliths and ZIF-8 particle were degassed at 150 °C for 2 hours before TGA measurements. A 2.7% weight loss was observed with the pure ZnO nanoparticles up to 700 °C. This is attributed to the removal of absorbed moisture and trapped atmospheric components as previously reported.[8] For the MOFs, no obvious weight loss was observed from neither the ZIF-8 monoliths nor ZIF-8 particles up to 350 °C indicating a thermally stable ZIF-8 structure, in line with previous reports.[9] From 350 - 700 °C, a weight loss of 64.4% and 63.7% is observed for the ZIF-8 monoliths and ZIF-8 particles, respectively, which can be attributed to the re-conversion of the ZIF-8 into ZnO. Considering the possible experimental errors, these values are in good agreement. Additionally, given the theoretical weight loss of 64.2% from a pure ZIF-8 to a ZnO, these provide further evidence for the full conversion of the ZnO FNN into a ZIF-8 monolith of *ca* 230 μm thick. The pure ZIF-8 structure of this 230 μm thick ZIF-8 monolith was also confirmed by XRD measurements as shown in Figure S22.

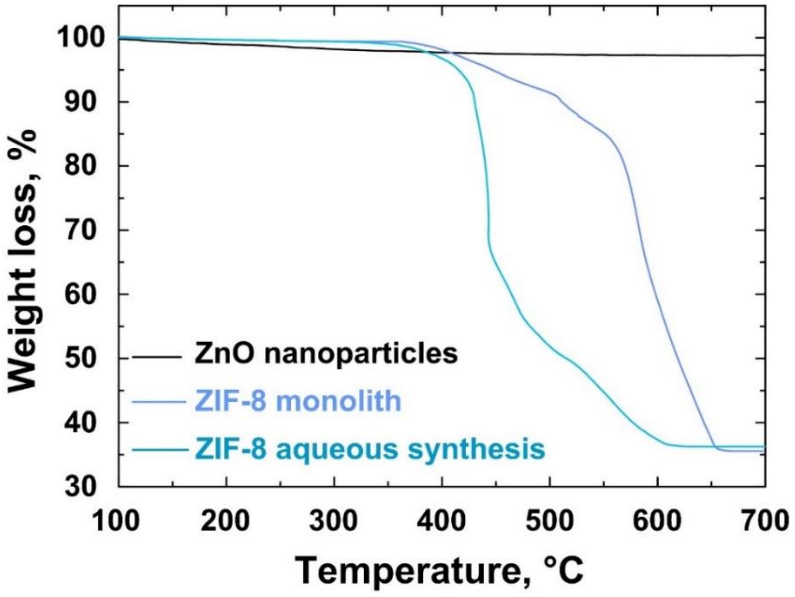

Figure S21 TGA characterization of the 230 μm thick ZIF-8 monolith. TGA curves of ZnO nanoparticles (black line), ZIF-8 monolith of *ca* 230 μm thickness (blue line), and ZIF-8 particles made via aqueous synthesis (dark cyan line).

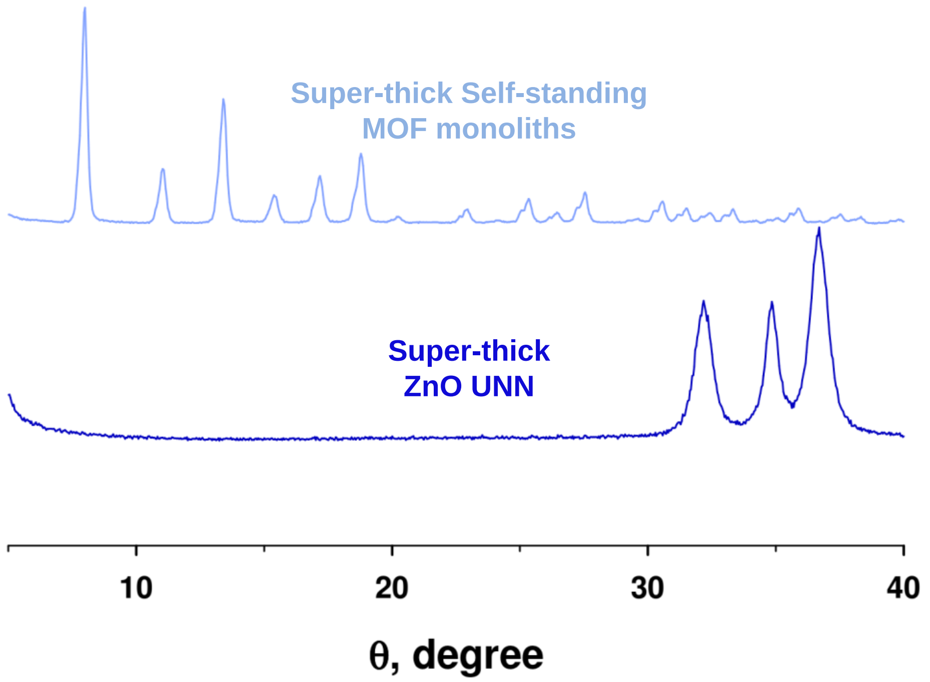

Figure S22 XRD analysis on the self-standing ZIF-8 monolith (*ca* 230 µm thick) before and after conversion. XRD patterns of ZnO FNN precursor before conversion (dark blue line) and after conversion into ZIF-8 monolith (*ca* 230 µm thick) (light blue line).

To demonstrate the designability of our proposed ceramic-to-MOF approach, ZnO FNNs with different designs have been prepared by either hand-drawing (Figure S23a-b) or introducing some existing templates (Figure S23c-d).

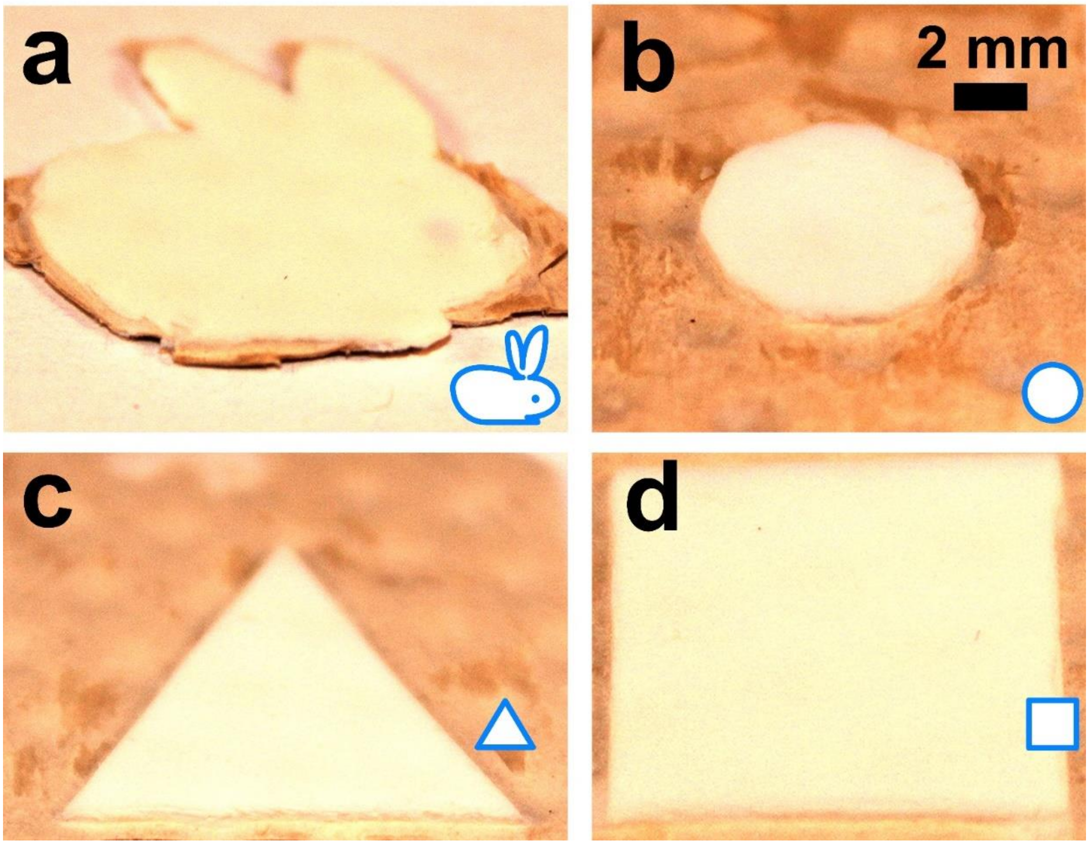

Figure S23 Optical images of *ca* 0.66 mm thick ZnO FNNs with different designs. a, A FNN rabbit. **b,** A FNN disk. **c,** A FNN triangle. **d,** A FNN square.

Fabrication of thinner monolithic self-standing MOF objects was achieved using our FNNs (Figure S24b, c, insets, self-standing ZIF-8 monolith of *ca* 12 μm in thickness). To acheive this, a SiO_2 FNN was used as the separation layer between the Si substrate and the ZnO FNN to facilitate the peeling off upon MOF conversion. More in detail, a double layer film consisting of a *ca* 30 μm thick ZnO FNN on top of a *ca* 20 μm SiO_2 FNN (Figure S24a, d and e) was prepared by sequential deposition of a SiO_2 and a ZnO nanoparticle aerosol. This double layered ZnO-SiO_2 FNN structure was converted into a MOF/SiO_2-FNN bilayer via the introduced approach at 150 °C resulting in a ZIF-8 monolith with thickness of *ca* 12 μm (Figure S24f-i), while the SiO_2 FNN remained almost unaffected (Figure S24e-f). A polymer blade thinner (< 20 μm) than the SiO_2 layer was adopted to suspend the ZIF-8 monoliths, resulting in their physically peel-off from substrate in the air (Figure S24b, c, insets). The detached ZIF-8 monoliths possess some unique properties including a square centimetre scale top surface area, medium-free transport and self-standing features (Figure S24b, c, insets, g-i). SEM images (Figure S24d-f) further confirm the integral MOF peeling off with only tiny amount of SiO_2 nanoparticles left on the MOF bottom side (Figure S24g-i). This guaranteed the detaching of MOF monoliths.

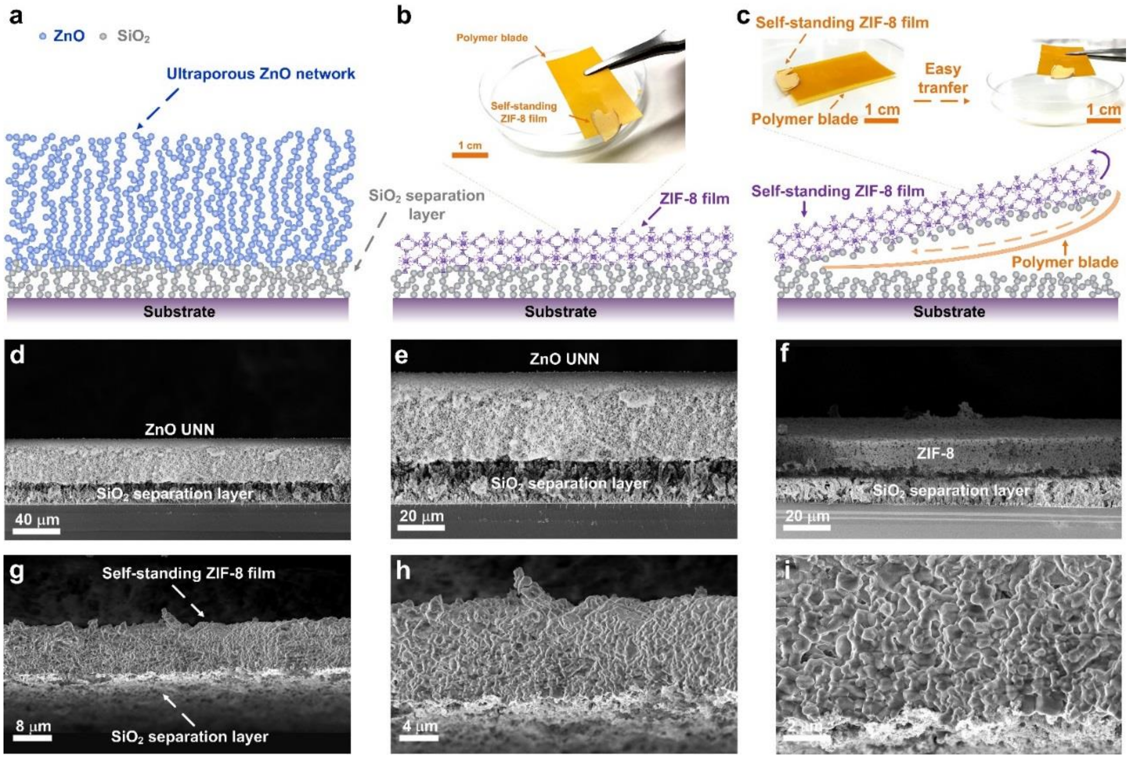

Figure S24 Schematic of the approach for fabrication of self-standing MOF monoliths, and SEM images of a representative 12 μm thick self-standing ZIF-8 monolith. a-c, Procedure applied to detach thin ZIF-8 monoliths from Si substrate and photos of an exemplary detached thin self-

standing ZIF-8 monoliths. **d-e,** SEM images of the two well-separated ZnO and SiO_2 FNNs. **f,** SEM images of **d** and **e** after conversion resulting in well-separated ZIF-8 and SiO_2 layers. **g-i,** SEM images of an exemplary self-standing thin ZIF-8 monolith of *ca* 12 μm. All samples were coated with a thin layer of Pt (*ca* 2 nm) to avoid charging.

A ZIF-8 monolith, with an identical thickness to the one employed as separator within the self-standing MOF interlayered Li-S cell (SMILC) batteries, has been prepared on a Si wafer. The as prepared ZnO FNN precursor is *ca* 67 μm in thickness. After conversion in presence of 2-MIM vapours at 150 °C for 18 hours, it resulted in a pure ZIF-8 monolith (*ca* 23 μm thick). The densification factor of *ca* 2.9 was also in line with our previous observations on film densification from a *ca* 7.1 μm thick ZnO FNN to ZIF-8 monolithic films (*ca* 2.8).

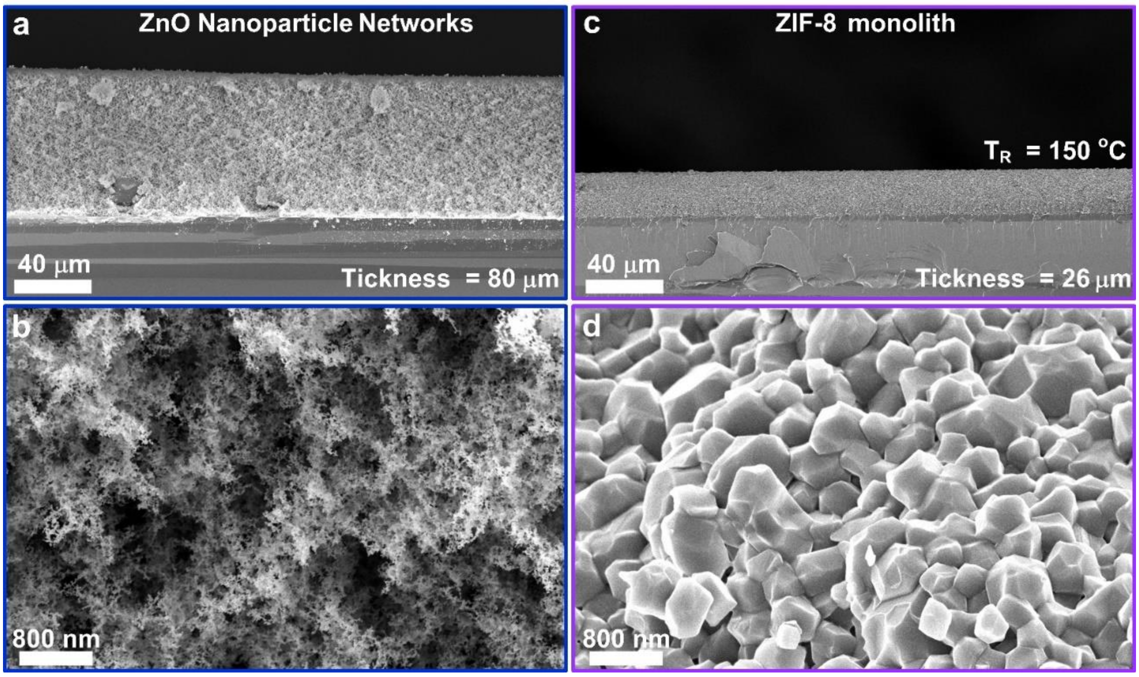

Figure S25 SEM images of the precursor ZnO FNN and the converted ZIF-8 membrane. a, SEM images of as prepared ZnO FNN precursor and (**b**) the corresponding higher magnification SEM image of **a**. **c,** SEM images of the converted pure ZIF-8 monolith from **a** and (**d**) the corresponding higher magnification SEM image of **c**. All samples were coated with a thin layer of Pt (*ca* 2 nm) to avoid charging.

Li-S system

Relying on its earth abundance and environmental friendliness,[10, 11] Sulfur is one of the most promising and visited cathode candidates for high-energy-density rechargeable batteries,[11-13] providing, amongst other benefits, a very high theoretical capacity of 1,675 $mAhg^{-1}$ and energy density of *ca* 2,500 $Whkg^{-1}$. The latter is about five times higher than that possible with conventional lithium-ion battery technology, based on conventional intercalation reactions.[11] As the limited maximal capacity and energy density of conventional lithium-ion battery technology are less able to meet the future user demands such as electrical vehicles and grid storage.[14, 15]

Further characterizations of the batteries

Given the electrochemical impedance spectra of the SMILC in the frequency range of 1.0 MHz to 0.1 Hz (**Figure 4g**), the high-frequency region is attributed to charge transfer resistance. It is noticeable that a significant semicircle shrinkage of about 94.6% occurred after the first cycle. The large initial impedance is tentatively attributed to the inhomogeneous sulfur/SuperP mixture in the cathode and incomplete wetting of MOF film and commercial separator before electrochemical cycling, while the slow decrease in impedance after the second cycles may result from the migration of active materials to more electrically favourable locations during discharge/charge process.[16]

While the poor performance of the bare commercial separators arise from their large pore size,[17] the poor cyclability of the cells with a supported MOFs membrane, made by FNN conversion, is tentatively attributed to the trapping of electrolyte and soluble LiPSs in the glass-fiber paper used as support. The overall worse performance and faster capacity decay of the cells with a wet-made MOFs membrane may arise from the lack of a continuous structure. The meso-macropores and cracks of these wet-made MOFs films may not be able to effectively reduce the shuttling of LiPSs, resulting in a similar capacity decay as in the case of the commercial separator. It is also supported by their poor CE (Supplementary Figure S26). In addition, the negligible absorption capability of ZIF-8 to LiPSs[18] as well as the structural uniformity of our monoliths ensures the low LiPSs loss and improved cycling stability.

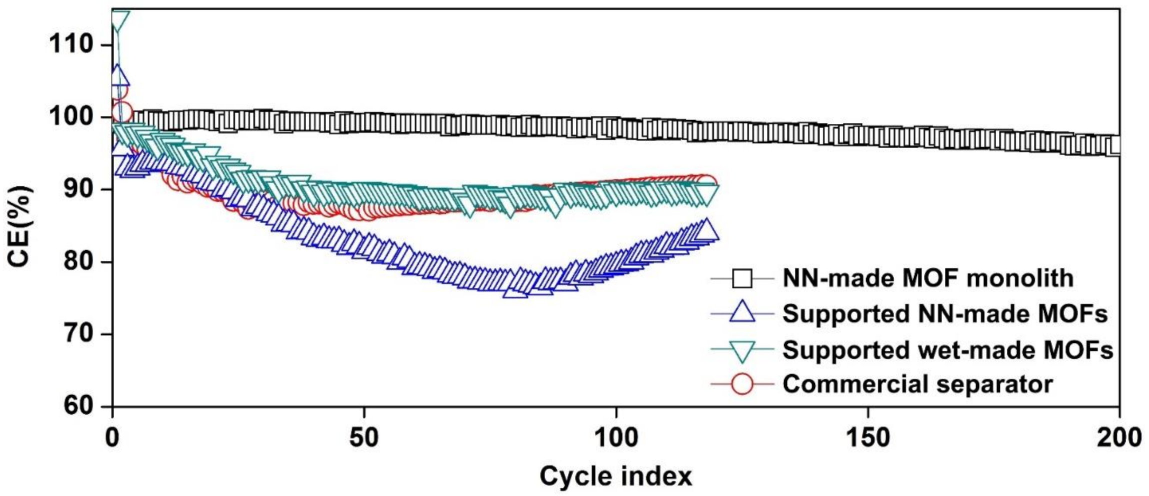

Figure S26 Comparison of Coulombic efficiencies of the batteries with the monolithic MOF membrane and other separators.

The ZIF-8 monolith alone has no obvious redox reaction with lithium in the voltage range of 1.5 V–2.8 V vs. Li/Li^+. This indicates that the majority of the SMILC capacity should be originated from the electrochemical reactions between lithium and sulfur/sulfides and the battery performance will only depends on the suppression of the shuttle effect of soluble LiPSs.[12, 15, 19]

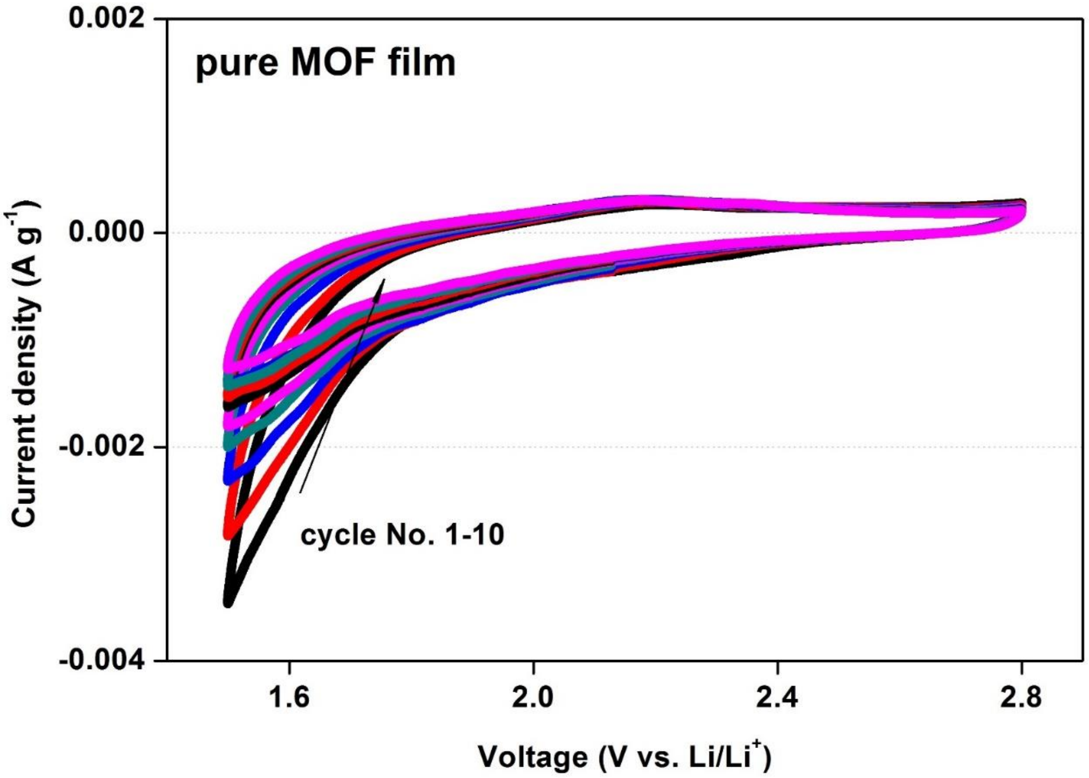

Figure S27 Cyclic voltammogram of a control cell with only ZIF-8 monolith as a cathode and Al foil as the current collector for the first 10 cycles.

Similar to the SMILC situation, there is also an obvious impedance drop from the initial state to the one after the 1st cycle. This suggests a decreased charge transfer resistance after electrochemical cycling. It is noticeable in Figure S28a that the Li–S battery without MOF interlayer has slightly smaller initial impedance than the SMILC (**Figure 4g**). This might be due to the incomplete wetting of the MOF membrane,[16, 17, 20] which resulted in higher charge transfer resistance before electrochemical cycling. Besides, as shown in Figure S28b, there are two semicircles in the high- and medium-frequency regions as well as an obvious increment of both semicircles from the 1st to the 5th cycle. These two phenomena did not happen for the SMILC. The two semicircles located in the high- and medium-frequency regions in the EIS plots could be attributed to the charge-transfer resistance and surface film impedance, respectively. It has been proposed that the additional surface film impedance could be originated from deposition of Li_2S/Li_2S_2 on Li metal surface due to severe LiPSs shuttle effect in the control battery. The increment of semicircles with cycle number could be due to the gradual growing of additional solid-electrolyte interface (SEI) layer by the deposition of Li_2S/Li_2S_2 on Li anode during cycling.

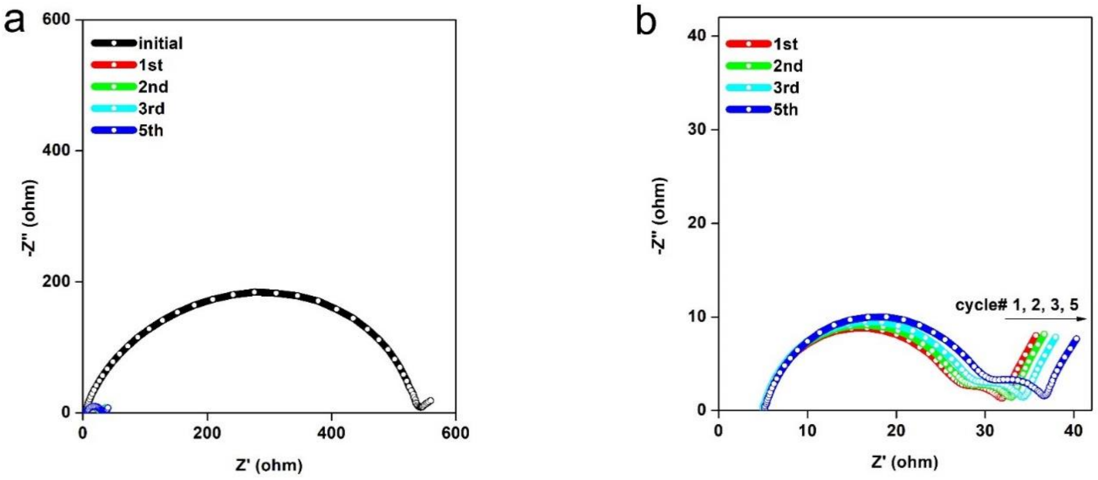

Figure S28 EIS plots of the control Li–S battery. a, EIS plots of the control Li–S battery at the initial state and cycles 1, 2, 3, and 5. **b,** A zoom-in view of EIS plots of cycles 1, 2, 3, and 5.

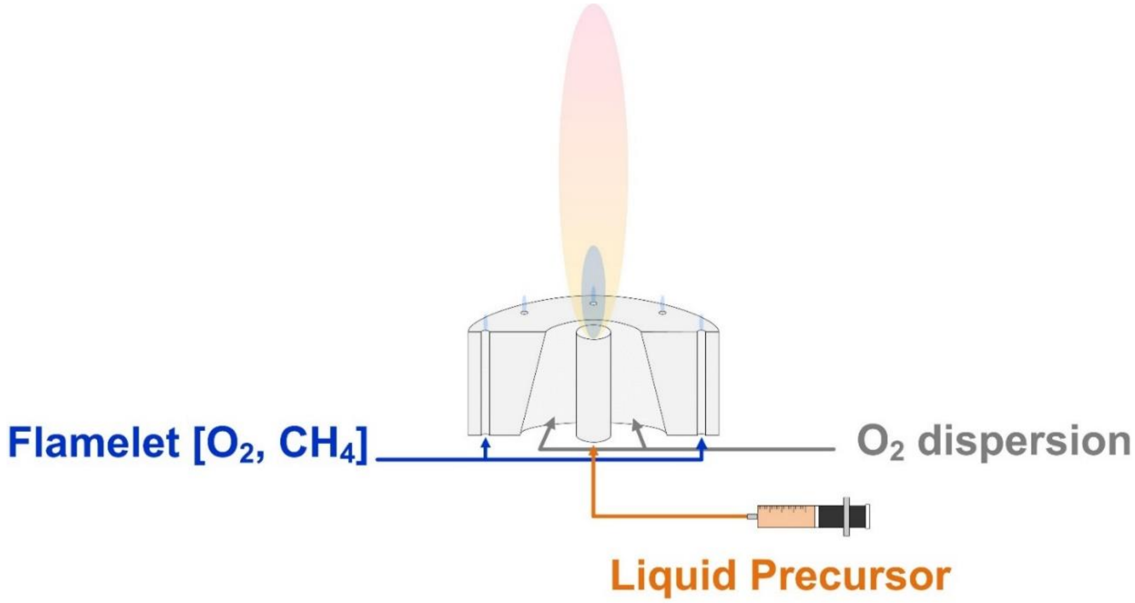

Figure S29 Schematic of the flame spray pyrolysis system used for the synthesis of the precursor ceramic nanoparticles and their self-assembly into a nanoparticle network (FNN).

Setup for reaction of the large ZnO FNN: A circular membrane (diameter: 15 cm) with ZnO FNN deposited on one side was placed in a stainless steel reaction chamber (16.5 × 16.5 × 2 cm of internal space, wall thickness of 1 cm, with a stainless steel cover lid of 20 × 20 × 1 cm), with the ZnO layer facing up. The chamber was pre-filled with 15 g of 2-MIM placed at the bottom of the internal space, leaving the middle area empty. The membrane was placed over a wire net (15 × 15 cm), in order to leave about 1 cm of space between the membrane and the bottom of the chamber, avoiding direct contact with the ligand powder. The chamber was sealed with a little amount of vacuum grease, to avoid leakage of ligand vapours, then placed in an oven at 120 °C for 18 hours. After the reaction time, the chamber was left to cool down to room temperature naturally before opening. The converted ZIF-8 membrane became more rigid after the conversion.

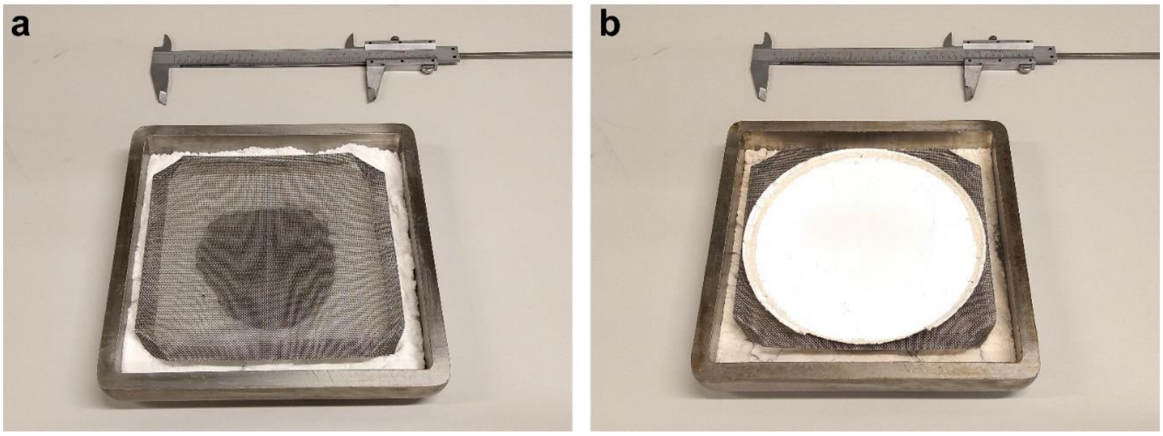

Figure S30 Optical images of a customized conversion reactor. a, Photograph of the customized conversion reactor filled with 2-MIM solid crystals before reaction. **b,** Photo of the customized conversion reactor opened after reaction with a 15 cm pure ZIF-8 membrane inside.

A Teflon lined stainless steel autoclave vial as shown in Figure S31 was adopted for our proposed approach.

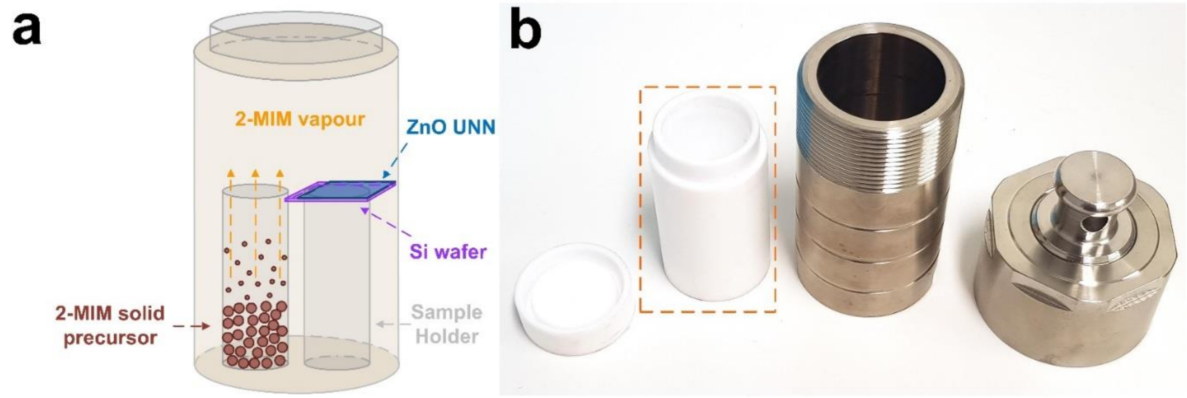

Figure S31 Teflon lined stainless steel autoclave vial. a, Schematic of the conversion setup. **b,** Photograph of setup accessories.

Calculations

The minimum required extrinsic porosity (P_{min}) for a full conversion from a ZnO FNN precursor to a ZIF-8 monolith is calculated using the following equation:

$$P_{min} = (1 - 1/\ R_{Exp}) \times 100\% \quad (1)$$

where R_{Exp} is the 17 folds' volumetric expansion factor from the wurtzite ZnO to ZIF-8 structure. This indicates, to achieve a full conversion, the minimum required extrinsic porosity from a ZnO FNN precursor lies at *ca* 94.12%.

The densification factor (C_d) from a ZnO FNN precursor to a ZIF-8 monolithic films at varied temperatures can be obtained via SEM analysis before and after reaction using the following equation:

$$C_d = T_{NN}/T_{MOF} \quad (2)$$

where T_{MOF} is the thickness of ZIF-8 monolithic films after vapour reaction at varied temperatures (Figure S14b, c, g, h and i, 90 – 150 °C) and T_{NN} is the thickness of ZnO FNN precursors before reaction (Figure S14a).

The extrinsic porosities of the converted ZIF-8 monolithic films at varied temperatures (90 – 150 °C) are obtained from calculations via two different approaches: (i) calculations (P_C) based on only densification factors of the obtained ZIF-8 monolithic films at different temperatures (90 – 150 °C) derived from SEM thickness analysis, and (ii) calculations (P_M) based on SEM and gravimetric analysis of Si wafer sections before and after the ZIF-8 monolithic film growth as reported elsewhere.[21]

P_C is determined using the following Equation (3):

$$P_C = [1 - (1 - P_{NN}) \times R_{Exp} \times C_d\ /100] \times 100\% \quad (3)$$

where P_{NN} is the extrinsic porosity of ZnO FNN precursors, R_{Exp} is the volumetric expansion factor from wurtzite ZnO to ZIF-8, and C_d is the densification factor from ZnO FNNs to ZIF-8 monolithic films.

P_M is determined using the following Equation (4):

$$P_M = [1 - (M_{Film\_MOF}/\rho_{MOF}/A_{Surface})/T_{SEM}] \times 100\% \quad (4)$$

where M_{Film} is the mass of a ZIF-8 monolithic film after conversion at a certain temperature (temperature ranging from 90 to 150 °C), ρ_{MOF} is the density of ZIF-8, $A_{Surface}$ is the surface area of the measured ZIF-8 monolithic films converted at each temperature, and T_{SEM} is the SEM thickness of ZIF-8 monolithic films converted at each temperature. Notably, the extrinsic porosity calculated using both approaches (i and ii) with over 20 batches are in line with each other as shown in Table S1, indicating a very good reproducibility and precisely controlled MOF extrinsic porosity via our proposed ceramic-to-MOF approach.

For instance, given the unvaried film thickness observed after vapour reaction at 90 °C, the extrinsic porosity of the resulting ZIF-8 monolithic film can be simply determined by the volumetric expansion of the primary crystal lattice from the wurtzite ZnO to ZIF-8 as followings:

$$P_C = [1 - (1 - P_{NN}/100) \times R_{Exp}] \times 100\% \qquad (5)$$

where R_{Exp} is the 17 folds' volumetric expansion factor from the wurtzite ZnO to ZIF-8. P_{NN} is the porosity of the ZnO FNN precursor.

According to the applied optimal ZnO FNN extrinsic porosity of 98% and the above Equation (5), it would result in a ZIF-8 monolithic film with an extrinsic porosity of 66% which is in line with the obtained extrinsic porosities of 68 ± 2% from our ZIF-8 monolithic film (at 90 °C) via calculation approach ii (Equation 4). In fact, Equation (5) is a simplification of Equation (3) where $C_d = 1$.

The effect of the reaction temperature on the resulting morphology and ZIF-8 extrinsic porosity can be attributed to the competition between Zn ion diffusion and reaction rate with 2-MIM. At low reaction temperatures the diffusivity of the Zn ions is limited resulting in a localized conversion. Increasing the temperature increases the Zn ion mobility and extent of reaction diffusion (RD);[22] as a result, the original network collapses into a more dense ZIF-8 monolith. The measured lower limit of the extrinsic porosity of *ca* 4% (at 150 °C) can be attributed to the closing of the meso and macro-pores, which slow the diffusion of the ligands into the inner part of the MOF monoliths, and may also result in trapping of some air pockets.

Further characterizations of the porous structure of these ZIF-8 monoliths were pursued by Brunauer-Emmett-Teller (BET) (Supplementary Table S1). These were carried out on both the ZIF-8 monoliths (*ca* 230 µm thick) made by the proposed ceramic-to-MOF approach, and on ZIF-8 particles of *ca* 250 nm in diameter synthesized using an aqueous synthesis. The BET SSA ($S_{F,BET}$) of the ZIF-8 monoliths (*ca* 230 µm thick) made by the ceramic-to-MOF conversion process was 1688 m^2g^{-1}, which is in agreement with the previous reports as well as the values obtained for control samples ($S_{P,BET}$) consisting of ZIF-8 particles of *ca* 250 nm in diameter, synthesized using an aqueous synthesis method (Supplementary Table S1).

All the values mentioned above and in **Calculations** section are shown in Table S1.

Table S1 Table of extrinsic porosity calculations and N_2 adsorption analysis.

ZIF-8 monolithic films' extrinsic porosities at varied temperatures (T_R) obtained from approach i (P_C) and approach ii (P_M), BET surface areas ($S_{P,BET}$) of ZIF-8 particles synthesized using an aqueous synthesis method and self-standing ZIF-8 monoliths of *ca* 230 µm ($S_{F,BET}$) converted at 150 °C.

T_R	P_C	P_M	BET	
			$S_{P,BET}$	$S_{F,BET}$
90 °C	66 ± 2%	68 ± 2%		
100 °C	43 ± 2%	46 ± 2%		
110 °C	27 ± 2%	26 ± 2%		
120 °C	11 ± 2%	11 ± 2%	1555 m^2g^{-1}	1688 m^2g^{-1}
150 °C	4 ± 2%	4 ± 2%		

Table S2 Performance comparison of Li–S batteries with MOF-based separators.

Separator	Morphology	S loading [mg cm^{-2}]	Initial capacity [mAh g^{-1}]	Cycle number (retention)	C-rate	Ref.
**Self-standing ZIF-8 film**	Free-standing monoliths	2.0 – 2.5	1276	200 (76%)	0.4C	**This work**
**Filtration-growth $Ni_3(HITP)_2$/PP**	sheets	-	~ 900	300 (65%)	0.5C	[23]
**Ce-UiO-66-BPDC/CNTs/PP**	powdery form	2.5	701.8	800 (49.8%)	1C	[24]
**Ce-MOF-808/CNTs/PP**			1021.8	800 (82.1%)		
**UiO-66-NH_2@SiO_2**	powdery form	0.5	1400	100 (42.9%)	0.1C	[25]
**Mn-BTC/PP**	powdery form	-	1430	80 (76.9%)	0.1C	[26]
**UiO-66/Nafion hybrid-coated**	powdery form	1.7	1127.4	200 (75.5%)	0.1C	[27]
**CNT@ZIF-30/PP**	carbon nanotubes	*ca* 1.2	1588.4	100 (54.8%)	0.2C	[28]
**CNT@ZIF-45/PP**			1374.2	100 (51.7%)		
**Interface-induced-growth $Ni_3(HITP)_2$/PP**	sheets	3.5	1224	100 (92%)	0.2C	[29]
			851	500 (84%)	1C	
**Y-FTZB/PP/CNT**	powdery form	*ca* 1.0	1101	300 (50.6%)	0.25C	[30]
**ZIF-7/PP/CNT**	powdery form	*ca* 1.0	1032	300 (43.8%)		
**ZIF-8/PP/CNT**	powdery form	*ca* 1.0	1025	300 (39.3%)		
**HKUST-1/PP/CNT**	powdery form	*ca* 1.0	989	300 (19.9%)		
**Micro/nanosized HKUST-1@GO/PP**	**powdery form**	**0.6 - 0.8**	**1126**	**500 (70.9%)**	**0.5C**	[31]

We have included a detailed table (Supplementary Table S2) comparing the performance of our as-obtained self-standing ZIF-8 separator with other MOF-based separators recently reported. When comparing with other MOF-based separators (Supplementary Table S2), our self-standing ZIF-8 separator shows a superior cycling performance (76% retention after 200 cycles) with a relatively high S loading of 2.0 – 2.5 mg cm^{-2} and an initial capacity of 1276 mAh g^{-1} under a comparatively high charging-discharging rate (0.4 C), see Supplementary Table S2. For

example, the interface-induced-grown $Ni_3(HITP)_2$/PP separators,[29] which could afford the highest S loading of 3.5 mg cm^{-2}, achieved lower initial capacities of 1224 and 851 mAh g^{-1} respectively. The CNT@ZIF-30/PP separators,[28] which afforded a higher initial capacity of 1588.4 mAh g^{-1}, had relatively low retention of 54.8% after only 100 cycles at a lower charging-discharging rate of 0.2 C. The Ce-MOF-808/CNTs/PP separators,[24] which presented a good cycling performance with a retention of 82.1%, however, experienced a *ca* 20% drop of initial capacity (1021.8 mAh g^{-1}). Therefore, taking all the different parameters into consideration (i.e. S loading, initial capacity, retention and charging-discharging rate), our self-standing ZIF-8 separators show among the best sets of properties (Supplementary Table S2). In addition, when comparing with other separators (e.g. powdery formed films or filtration-grown sheets), our self-standing ZIF-8 separators could prevent the leaking of LiPSs and enhance the battery performance. A disadvantage of thin ZIF-8 monolithic membranes is the typical brittleness of MOF materials, which results in a reduced mechanical robustness than other membranes made of more robust materials (e.g. CNT or GO). However, the optimization of the ZIF-8 membrane thickness to 23 μm resulted in sufficient mechanical properties for both the assembly of the battery and the long-term cycling test.

The effect of pressure on the ZIF-8 film

With respect to the effect of pressure in the ZnO to ZIF-8 (Zn(2-MIM) with sod topology) conversion, we have now calculated the saturation pressure of 2-MIM at all tested temperatures including 90, 100, 110, 120 and 150 °C using Antoine's Equation firstly, in line with previous reports.[2,32]

$$\log p = A - \frac{B}{T}$$

Where p is the saturation pressure (here P_{sat}) in Pa, T is the temperature in Kelvin (K), A and B are Antoine's constants of 2-MIM (A = 14.30 and B = 4608.90[32]), respectively. After calculating the saturation pressure of 2-MIM at different temperatures, the amount of solid MOF ligand required to create a vapour of saturation pressure has been estimated using ideal gas equation:

$$\mathrm{PV} = \mathrm{nRT}$$

where P used in the equation can be expressed as saturation pressure (P_{sat}), V is the volume of our reaction chamber (i.e. 80 ml), R is the ideal gas constant (8.314 J mol^{-1} K^{-1}), T is the temperature in Kelvin (K) and n is the amount of 2-MIM in moles, respectively. The calculated results are shown in the following table:

Table S3. Saturation vapour pressure (P_{sat}) and required mass of 2-MIM ligand at different temperatures.

T (temperature)/°C	P_{sat} (saturation pressure)/Pa	m (mass required)/mg
90	40.11	0.09
100	87.84	0.19
110	184.64	0.38
120	373.70	0.75
150	2536.62	4.74

As shown above, a tiny amount of 2-MIM is enough to reach the vapour saturation pressure at all listed temperatures. In our experiments, the typical ZnO mass is around 0.6 to 0.8 mg, thus the theoretical stoichiometric amount of 2-MIM needed is in the 1.2-1.6 mg range for the full conversion to ZIF-8. However, in our experiments, we used an amount of 2-MIM in the range of several hundred milligrams to reach and maintain $P>P_{sat}$ throughout all the gas phase conversions.

According to the accuracy related to the amount of 2-MIM needed to reach P_{sat} at different temperatures, we selected 150 °C as a conversion temperature to examine the effect of the ligand vapour pressure. At 150 °C , by decreasing the amount of 2-MIM from 100 mg to 2 mg a ligand vapour pressure of $P_{2mg@150}$=1070.90 Pa was obtained; this pressure is less than half that of the saturation pressure ($P_{2mg@150}$=0.42 $P_{sat@150}$). Notably, upon the standard 18 h reaction time at 150 °C at this lower ligand pressure ($P_{2mg@150}$=1070.90 Pa), the ZnO was not fully converted to ZIF-8 (Figure S32, below). As such, our data show that for $P \geq P_{sat}$, an excess of 2-MIM does not influence the ZnO to ZIF-8 conversion. Instead, for P<Psat only a partial conversion from ZnO to ZIF-8 is observed.

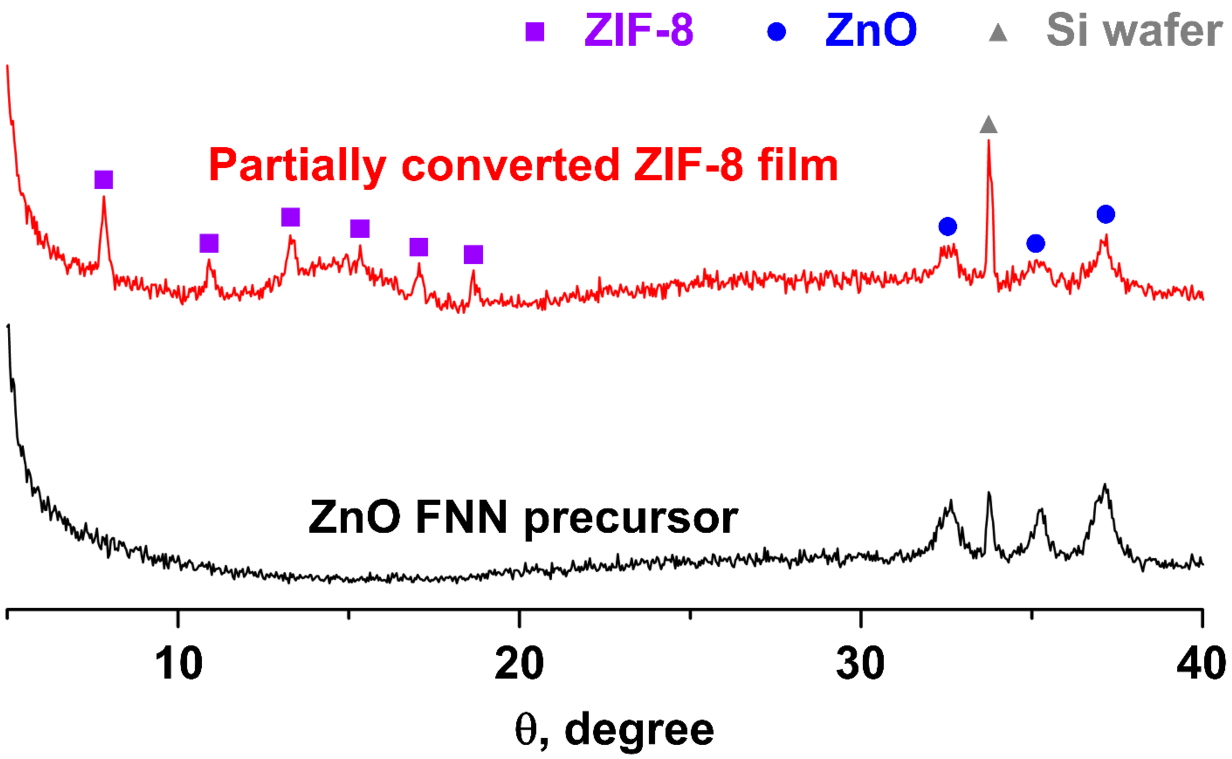

Figure S32 XRD of partially converted FNN precursor at 150 °C under an unsaturated pressure of 1070.90 Pa.

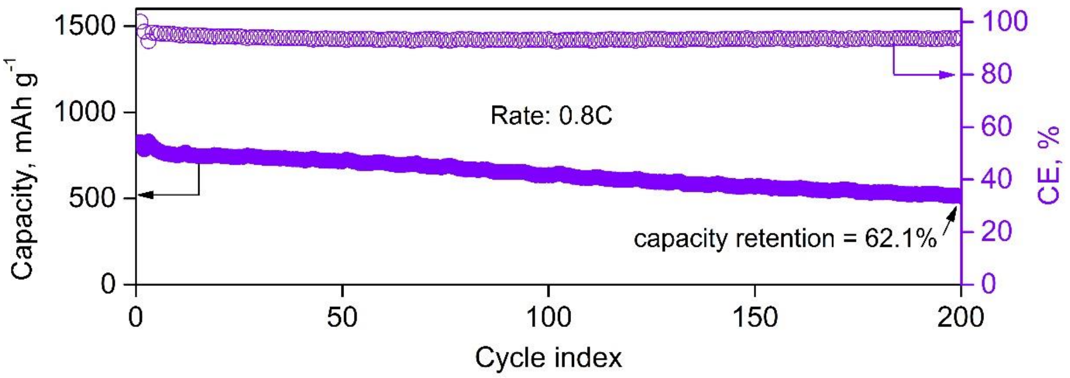

Figure S33 Cycling performance of self-standing ZIF-8 separator at a discharge rate of 0.8 C.

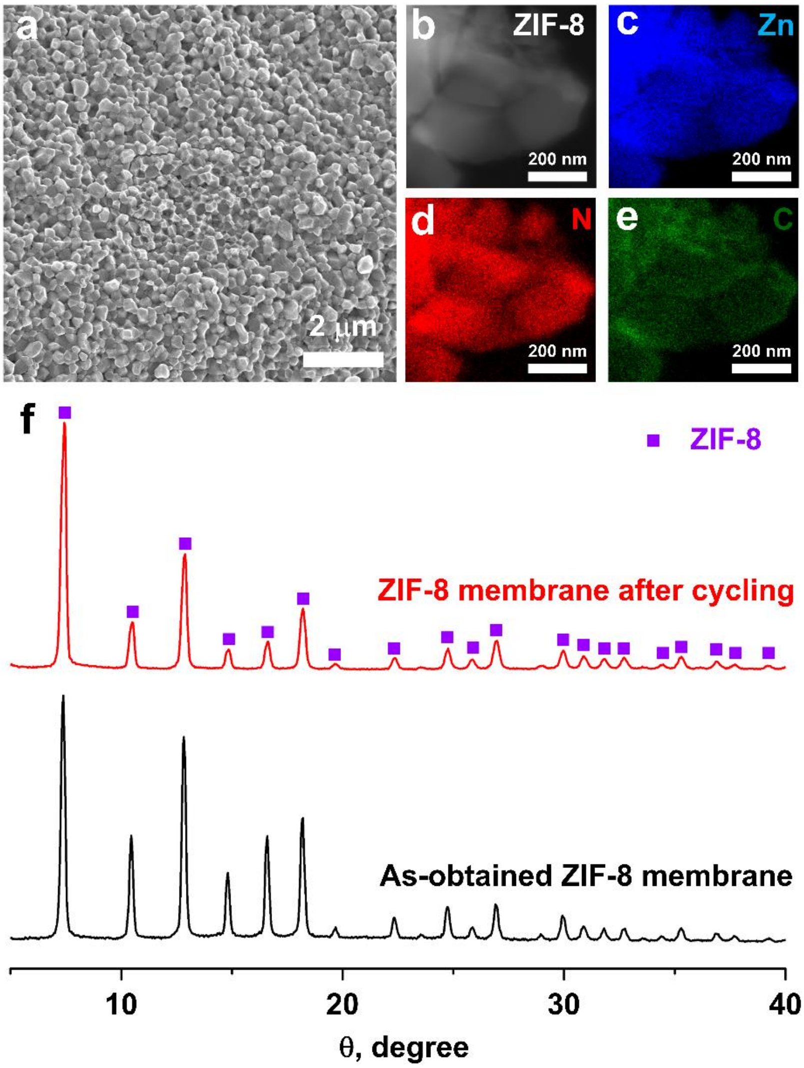

Figure S34 Post battery characterizations. a, Cross-sectional SEM images of self-standing ZIF-8 membrane after 200 cycles. **b-e,** HRTEM images of ZIF-8 particles detached from self-standing ZIF-8 membrane after 200 cycles (**b**) and elemental mapping (**c-e**). Zn, N and C are presented in blue, red and green, respectively. **f,** X-Ray diffraction pattern of ZIF-8 membrane before (black line) and after 200 cycles (red line).

References

[1] S. Tanaka, K. Kida, T. Nagaoka, T. Ota, Y. Miyake, ***Chemical Communications*** 2013, 49, 7884.
[2] I. Stassen, M. Styles, G. Grenci, Hans V. Gorp, W. Vanderlinden, Steven D. Feyter, P. Falcaro, D. D. Vos, P. Vereecken, R. Ameloot, ***Nature Materials*** 2016, 15, 304.
[3] A. Tricoli, S. E. Pratsinis, ***Nature Nanotechnology*** 2010, 5, 54.
[4] R. Bo, N. Nasiri, H. Chen, D. Caputo, L. Fu, A. Tricoli, ***ACS applied materials & interfaces*** 2017, 9, 2606.
[5] M. Jian, B. Liu, R. Liu, J. Qu, H. Wang, X. Zhang, ***RSC Advances*** 2015, 5, 48433.
[6] P. C. Rowlette, C. A. Wolden, ***ACS applied materials & interfaces*** 2009, 1, 2586.
[7] Z. Fusco, M. Rahmani, R. Bo, R. Verre, N. Motta, M. Käll, D. Neshev, A. Tricoli, ***Advanced Materials*** 2018, 30, 1800931.
[8] N. Nasiri, R. Bo, F. Wang, L. Fu, A. Tricoli, ***Advanced Materials*** 2015, 27, 4336.
[9] C. Avci, I. Imaz, A. Carné-Sánchez, J. A. Pariente, N. Tasios, J. Pérez-Carvajal, M. I. Alonso, A. Blanco, M. Dijkstra, C. López, ***Nature Chemistry*** 2018, 10, 78.
[10] Y. X. Yin, S. Xin, Y. G. Guo, L. J. Wan, ***Angewandte Chemie International Edition*** 2013, 52, 13186.
[11] P. G. Bruce, S. A. Freunberger, L. J. Hardwick, J.-M. Tarascon, ***Nature Materials*** 2012, 11, 19.
[12] A. Manthiram, Y. Fu, S.-H. Chung, C. Zu, Y.-S. Su, ***Chemical Reviews*** 2014, 114, 11751.
[13] Z. W. Seh, Y. Sun, Q. Zhang, Y. Cui, ***Chemical Society Reviews*** 2016, 45, 5605.
[14] V. Etacheri, R. Marom, R. Elazari, G. Salitra, D. Aurbach, ***Energy & Environmental Science*** 2011, 4, 3243.
[15] M. Armand, J.-M. Tarascon, Nature 2008, 451, 652; B. Dunn, H. Kamath, J.-M. Tarascon, ***Science*** 2011, 334, 928.
[16] Y.-S. Su, A. Manthiram, ***Nature Communications*** 2012, 3, 1166.
[17] S. Bai, X. Liu, K. Zhu, S. Wu, H. Zhou, ***Nature Energy*** 2016, 1, 16094.
[18] Y. Zang, F. Pei, J. Huang, Z. Fu, G. Xu, X. Fang, ***Advanced Energy Materials*** 2018, 1802052.
[19] Y. Yang, G. Zheng, Y. Cui, ***Chemical Society Reviews*** 2013, 42, 3018; Z. W. Seh, W. Li, J. J. Cha, G. Zheng, Y. Yang, M. T. McDowell, P.-C. Hsu, Y. Cui, ***Nature Communications*** 2013, 4, 1331; A. Manthiram, S. H. Chung, C. Zu, ***Advanced Materials*** 2015, 27, 1980.
[20] G. Zhou, L. Li, D. W. Wang, X. y. Shan, S. Pei, F. Li, H. M. Cheng, ***Advanced Materials*** 2015, 27, 641; Y. Mao, G. Li, Y. Guo, Z. Li, C. Liang, X. Peng, Z. Lin, ***Nature Communications*** 2017, 8, 14628; S. H. Chung, A. Manthiram, ***Advanced Materials*** 2014, 26, 1360.
[21] A. Tricoli, M. Graf, F. Mayer, S. Kuühne, A. Hierlemann, S. E. Pratsinis, ***Advanced Materials*** 2008, 20, 3005.
[22] I. R. Epstein, B. Xu, ***Nature Nanotechnology*** 2016, 11, 312.
[23] H. Chen, Y. Xiao, C. Chen, J. Yang, C. Gao, Y. Chen, J. Wu, Y. Shen, W. Zhang, S. Li, ***ACS applied materials & interfaces*** 2019.
[24] X.-J. Hong, C.-L. Song, Y. Yang, H.-C. Tang, G.-H. Li, Y.-P. Cai, H. Wang, ***ACS nano*** 2019.
[25] S. Suriyakumar, A. M. Stephan, N. Angulakshmi, M. H. Hassan, M. H. Alkordi, ***Journal of Materials Chemistry A*** 2018, 6, 14623.
[26] S. Suriyakumar, M. Kanagaraj, M. Kathiresan, N. Angulakshmi, S. Thomas, A. M. Stephan, ***Electrochimica Acta*** 2018, 265, 151.
[27] S. H. Kim, J. S. Yeon, R. Kim, K. M. Choi, H. S. Park, ***Journal of Materials Chemistry A*** 2018, 6, 24971.
[28] F. Wu, S. Zhao, L. Chen, Y. Lu, Y. Su, Y. Jia, L. Bao, J. Wang, S. Chen, R. Chen, ***Energy Storage Materials*** 2018, 14, 383.
[29] Y. Zang, F. Pei, J. Huang, Z. Fu, G. Xu, X. Fang, ***Advanced Energy Materials*** 2018, 8, 1802052.
[30] M. Li, Y. Wan, J.-K. Huang, A. H. Assen, C.-E. Hsiung, H. Jiang, Y. Han, M. Eddaoudi, Z. Lai, J. Ming, ***ACS Energy Letters*** 2017, 2, 2362.
[31] S. Bai, X. Liu, K. Zhu, S. Wu, H. Zhou, ***Nature Energy*** 2016, 1, 16094.
[32] P. Jiménez, M. Roux, C. Turrión, The Journal of Chemical Thermodynamics 1992, 24, 1145.
